# Initiating Electron Transfer in Doubly Curved Nanographene Upon Supramolecular Complexation of C_60_

**DOI:** 10.1002/anie.202112834

**Published:** 2021-12-29

**Authors:** Simon Zank, Jesús M. Fernández‐García, Anton J. Stasyuk, Alexander A. Voityuk, Marcel Krug, Miquel Solà, Dirk M. Guldi, Nazario Martín

**Affiliations:** ^1^ Department of Chemistry and Pharmacy Friedrich-Alexander-Universität Egerlandstrasse 3 91058 Erlangen Germany; ^2^ Departamento de Química Orgánica I Facultad de Ciencias Químicas Universidad Complutense de Madrid Avd. de la Complutense, S/N 28040 Madrid Spain; ^3^ Institut de Química Computacional and Departament de Química Universitat de Girona C/ Maria Aurèlia Capmany 69 17003 Girona Spain; ^4^ Institució Catalana de Recerca i Estudis Avancats (ICREA) 08010 Barcelona Spain; ^5^ IMDEA-Nanociencia C/ Faraday, 9, Campus de Cantoblanco 28049 Madrid Spain

**Keywords:** curved nanographenes, DFT, electron transfer, fullerene, supramolecular complexation

## Abstract

The formation of supramolecular complexes between C_60_ and a molecular nanographene endowed with both positive and negative curvatures is described. The presence of a corannulene moiety and the saddle shape of the molecular nanographene allows the formation of complexes with 1:1, 1:2, and 2:1 stoichiometries. The association constants for the three possible supramolecular complexes were determined by ^1^H NMR titration. Furthermore, the stability of the three complexes was calculated by theoretical methods that also predict the photoinduced electron transfer from the curved nanographene to the electron acceptor C_60_. Time‐resolved transient absorption measurements on the ns‐time scale showed that the addition of C_60_ to **NG‐1** solutions and photo‐exciting them at 460 nm leads to the solvent‐dependent formation of new species, in particular the formation of the one‐electron reduced form of C_60_ in benzonitrile was observed.

## Graphical abstract

Concave–convex interactions between negatively and positively curved molecular nanographene with C_60_ allow the formation of complexes with 1:1, 1:2, and 2:1 stoichiometries. Theoretical calculations predict the photoinduced electron transfer from the curved nanographene to the electron acceptor C_60_. Transient absorption spectroscopy confirms solvent dependent electron transfer after photo‐excitation.

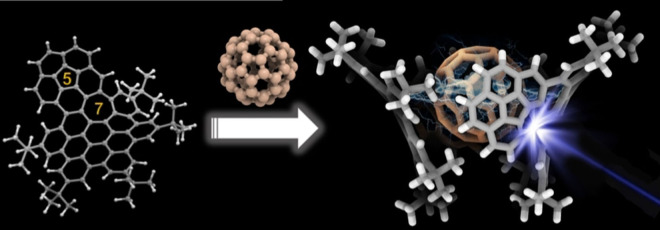

## Introduction

Graphene quantum dots are electron confined flakes of graphene with dimensions usually in the 3–20 nm range. They are considered as a singular family of zero dimensional (0D) carbon‐based materials, since, in contrast to pristine graphene, these nanomaterials are luminescent with a non‐zero band gap stemming from quantum confinement and edge effects.^[1]^ By virtue of such unique electronic features, they render outstanding materials for optoelectronic devices,^[2]^ energy storage systems,^[3]^ perovskite solar cells,^[4]^ and biomedical applications.^[5]^

**Figure 1:**
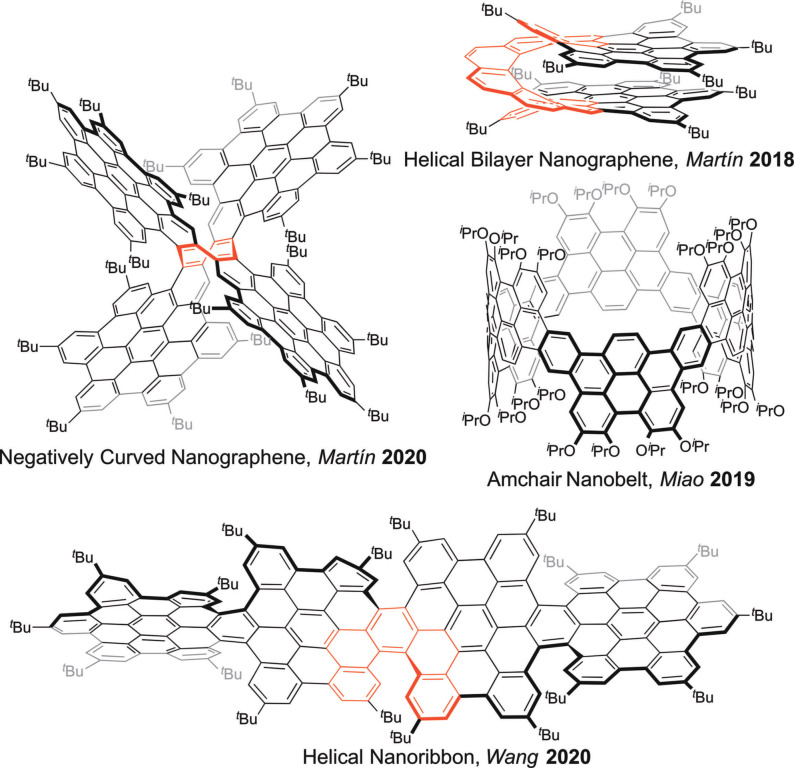
Recent examples of nanographenes with different topologies synthesized by bottom‐up approach.

In recent years, bottom‐up approaches have enabled solution‐phase syntheses of these materials in a step‐by‐step fashion. This has resulted in an accurate control over size, morphology and, therefore, on‐demand fine‐tuning of the properties.^[6]^ Molecular nanographenes with a wide range of shapes, namely bilayers,^[7]^ belts,^[8]^ ribbons,^[9]^ propellers,^[10]^ helical,^[11]^ planar^[12]^ and curved,^[13]^ have been prepared by using controlled bottom‐up approaches (Figure 1).

Synthetic implementation of defects such as non‐hexagonal rings into the 2D honey‐comb lattice endows Gaussian curvature to molecular nanographenes. Introducing five‐ or four‐membered rings leads to positive Gaussian curvature affording bowl shape morphologies. In contrast, inserting rings larger than hexagons involves the formation of negative Gaussian curvature and implies saddle shape structures. No doubt, the co‐existence of both curvature motifs in molecular nanographenes is extraordinary. Thus, the syntheses and properties of several nanographenes with a combined bowl and saddle shape have been reported in the literature.^[14]^

**Figure 2:**
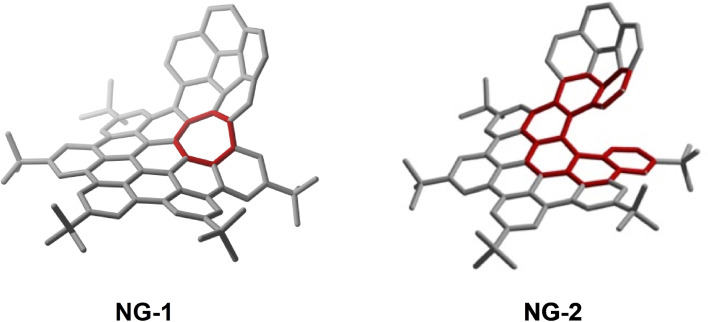
π‐extended corannulene‐based curved nanographenes **NG‐1** and **NG‐2**.

Recently, our research group has described the synthesis of corannulene‐based nanographenes featuring both positive and helical or negative curvatures.^[15]^ This procedure is based on the π‐extension of positively curved bromocorannulene by sequential Sonogashira, Diels–Alder, and Scholl reactions. Oxidant‐dependent and selective Scholl closure of a seven‐membered ring leads to a positively and simultaneously negatively curved molecular nanographene **NG‐1** or to a positive‐curved helical molecular nanographene **NG‐2** when the ring closure does not take place (Figure 2).

Curvature of nanographene **NG‐1** renders it a unique host for the complexation of fullerenes, driven by concave‐convex interactions. As a matter of fact, complexation by any negatively curved nanographenes remains poorly explored. In addition, the electron‐accepting nature of C_60_ gives rise to discernible electronic communication with, for example, suitable electron donors, especially under light irradiation.^[16]^

In the current contribution, we report on results regarding static and dynamic interactions between **NG‐1** and C_60_. The combination of these two compounds creates a system which is solely based on carbon and hydrogen. Furthermore, theoretical calculations underpin the stability of the different complexes and predict that photoinduced electron transfer occurs in the picosecond timescale. Nanosecond time‐resolved assays pointed out the tunability of the dynamic processes. In polar benzonitrile, electron transfer from the curved nanographene to C_60_ leading to the formation of the respective radical‐ion pair is observed. In less polar chlorobenzene, the radical ion pair cannot be stabilized and triplet excited state energy transfer from **NG‐1** to C_60_ remains.

## Results and Discussion

As aforementioned, curved **NG‐1** and **NG‐2** have been synthesized by Sonogashira–Diels–Alder–Scholl sequential reactions.^[16]^ The aim of this work is focused on the supramolecular complexation of **NG‐1** with C_60_ by taking advantage of their respective concave and convex geometries.^[17]^

**Figure 3:**
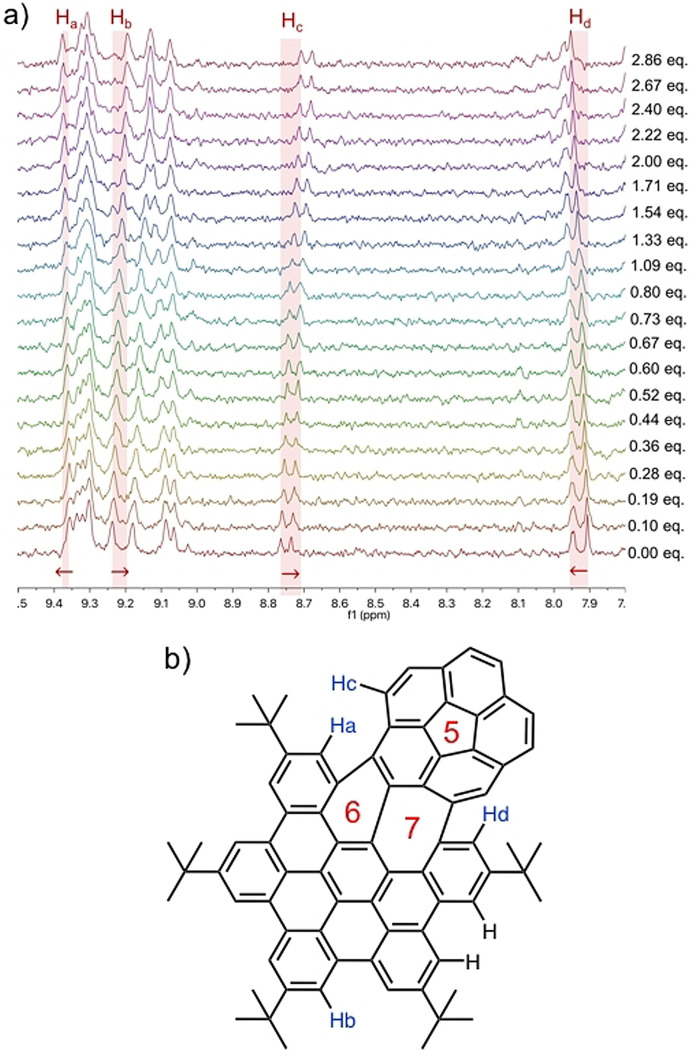
a) ^1^H NMR spectra of **NG‐1** with sequential additions of C_60_. b) Signal assignment of **NG‐1** by 2D NMR experiments.

Experimental corroboration for the complexation of, for example, curved **NG‐1** and C_60_ was performed by ^1^H‐NMR titration. In an NMR sample tube, a 5×10^−4^ M [D_8_]toluene solution of **NG‐1** was prepared. Sequential additions of a solution of C_60_ (2×10^−3^ M) and **NG‐1** (5×10^−4^ M) resulted in ^1^H‐NMR spectra, which are shown in Figure 3 a. Several signals shift down‐field and prompt to the successful formation of **NG‐1**⋅C_60_. In this way, signals at *δ*=9.36 (H_a_) and *δ*=7.91 (H_d_) underwent shifts to lower fields by Δ*δ* H_a_=−0.02 ppm and Δ*δ* H_d_=−0.05 ppm, respectively. Signals at *δ*=9.24 (H_b_) and *δ*=8.76 (H_c_) experienced shifts to higher fields Δ*δ* H_b_=0.04 ppm and Δ*δ* H_c_=0.05 ppm. 2D‐NMR experiments were used to assign these signals (Figure 3 b). H_a_, H_c_, and H_d_ match those protons, which are located at the interface between the corannulene and the rest of the π‐system. In other words, in close proximity to the seven‐membered ring, where the curvature is most significant, and where concave‐convex interactions between **NG‐1** and C_60_ are strongest.

The association constants have been calculated taking three different complex stoichiometries, namely 1:1 (**NG‐1**⋅C_60_), 1:2 (**NG‐1**⋅(C_60_)_2_), and 2:1 ((**NG‐1**)_2_⋅C_60_), into account. Considering a 1:1 stoichiometry in **NG‐1**⋅C_60_, complexes are formed between the π‐system containing the concave surface of corannulene and C_60_ by means of concave‐convex interactions. Here, the proton‐shifts for H_a_, H_b_, H_c_, and H_d_ fit with the model with a low value of Sum of Squared Residuals SSR_1:1_=1.0024×10^−4^. From this, we conclude a rather tight fit of the model with the data. In this model, the calculated association constant is *K*_a_=1.17×10^3^ M^−1^ and the mole fraction of **NG‐1**⋅C_60_ is 0.5 when 2.2 equivalents of C_60_ were added (Figure S2).

For **NG‐1**⋅(C_60_)_2_, additionally to the aforementioned interactions in **NG‐1**⋅C_60_, a second set of concave‐convex interactions occur at, however, the opposite face of **NG‐1**. It is based on the negative curvature that confers the saddle‐shape of **NG‐1**. CH‐π interactions between ^*t*^Bu groups of **NG‐1** and C_60_ are likely to play an important role. The data fit brings SSR_1:2_=5.42×10^−5^, which is somewhat better than the 1:1 stoichiometry fittings. The calculated association constants are *K*_a1_=1.69×10^3^ M^−1^ and *K*_a2_=1.16×10^3^ M^−1^. Lastly, a 2:1 stoichiometry model was studied. (**NG‐1**)_2_⋅C_60_ implies a complexation between the π‐extended concave face of corannulene of two molecules of **NG‐1** and one C_60_. Overall, the fullerene remains inside the nanographene cage (SSR_2:1_ is 5.14×10^−5^). From the calculated association constants of *K*_a1_=1.71×10^3^ M^−1^ and *K*_a2_=3.17×10^3^ M^−1^ we derive the coexistence of three plausible species **NG‐1**⋅C_60_, **NG‐1**⋅(C_60_)_2_, and (**NG‐1**)_2_⋅C_60_ in almost the same concentration when about 1.5 equivalents of C_60_ were added (Figure S4).

**Figure 4:**
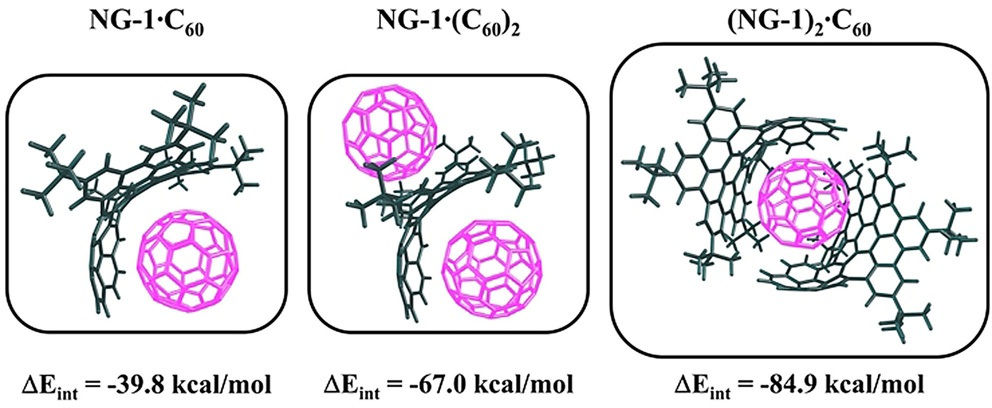
Optimized structures and interaction energies of the most stable conformers obtained at the BLYP‐D3(BJ)/def2‐TZVP//BLYP‐D3(BJ)/def2‐SVP level of theory.

For **NG‐1**⋅C_60_, two conformers are possible. The first isomer is formed by non‐covalent interactions between C_60_ and the concave surface of corannulene, whereas in the second isomer C_60_ interacts with the saddle‐shaped surface on the opposite side of **NG‐1**. The latter is 7.9 kcal mol^−1^ less stable than the former according to our calculations (Figure S5, SI). In **NG‐1**⋅(C_60_)_2_ complex, each side of **NG‐1** interacts with one C_60_, thus only one isomer is possible, while for (**NG‐1**)_2_⋅C_60_ multiple isomers with different mutual orientations of the fragments we found. Figure S5, SI, depicts several isomers found for these complexes. The relative stability of the conformers of **NG‐1**⋅C_60_, **NG‐1**⋅(C_60_)_2_, and (**NG‐1**)_2_⋅C_60_ complexes in the ground state (GS) was assessed by DFT calculations at the BLYP‐D3(BJ)/def2‐SVP level of theory^[18]^ (see SI for details). The structures of the most stable conformers are shown in Figure 4.

To estimate the stability of each complex, the interaction energies (Δ*E*_int_) between **NG‐1** and C_60_ were computed. For the most stable conformers of **NG‐1**⋅C_60_, **NG‐1**⋅(C_60_)_2_, and (**NG‐1**)_2_⋅C_60_, Δ*E*_int_ is found to be −39.83, −66.98, and −84.94 kcal mol^−1^, respectively. We infer that these interaction energies are somewhat overestimated. For **NG‐1**⋅C_60_, accounting for the basis set superposition error (BSSE) reduces the interaction energy by ca. 2 kcal mol^−1^, whereas the use of hybrid and range corrected functionals results in lower stabilization energies, which vary in the range from 5 to 9 kcal mol^−1^, (Table S2, SI). Similar to previously reported carbon‐rich complexes,^[19]^ the interaction energy in (**NG‐1**)_2_⋅C_60_ is superadditive. In particular, the total stabilization energy is 5 kcal mol^−1^ larger than the sum of interaction energies in two **NG‐1**⋅C_60_ (Figure S6, SI).

Taking into account the electron acceptor properties of C_60_, we looked at the charge separation between **NG‐1** and C_60_ in the ground state. The population analysis provides no evidence for significant charge transfer between the fragments (Table S3, SI). In addition, we performed an energy decomposition analysis (EDA), which enables to divide the interaction energy into four components: electrostatic (Δ*E*_elstat_), Pauli repulsion (Δ*E*_Pauli_), orbital (Δ*E*_oi_), and dispersion correction (Δ*E*_disp_) (Table S4, SI). Binding forces in these complexes were found to be similar. The largest contribution to the attraction between the two constituents is the dispersion term (around 60 %), which is followed by the electrostatic term (26–29 %) and the orbital interaction term (13–15 %). The topology of the non‐covalent interactions was described using the NCI calculations^[20]^ (Figures S7 and S8, SI).

The simulation of the lowest 80 excited states was carried out by the TDA‐DFT method with the CAM‐B3LYP‐D3(BJ)/def2‐SVP//BLYP‐D3(BJ)/def2‐SVP Scheme.[^18c^, ^21^] To characterize the excited states, the complexes were divided into **NG‐1** and C_60_ fragments (two in the case of **NG‐1**⋅C_60_ or three in the cases of **NG‐1**⋅(C_60_)_2_ and (**NG‐1**)_2_⋅C_60_. Within this Scheme, three types of excited states can be identified: (1) locally excited (LE) states, in which the excitation is mainly localized only on a single fragment and the degree of charge separation is less than 0.1 ***e*** (CS value <0.1 ***e***); (2) charge separated (CS) states with a significant amount of electron density transferred between the fragments (CS value >0.8 ***e***) and (3) mixed states with a significant contribution of both LE and CS (0.1 ***e*** **<** CS value <0.8 ***e***).

In the gas phase, the excitation energies range from 2.46 to 4.25 eV (Table S5, SI). In all complexes, the LE state on C_60_ (LE_1_) has the lowest energy. The LE states with an exciton on **NG‐1** (LE_2_) are 0.58–0.65 eV higher in energy. Among the 80 lowest excited states, we found only CS states that correspond to the electron transfer from **NG‐1** to C_60_. The energy of the lowest CS state varies slightly from 2.65 to 2.72 eV depending on the stoichiometry of the complex. The direct population of the CS states due to light absorption is unlikely due to their low oscillator strength (f) of <0.001. However, the CS states can be generated by the decay of LE states. The highly absorptive states, with an oscillator strength ranging from 0.32 to 0.74 are found in the complexes at 3.83–3.89 eV (Table S5, SI). The Kohn–Sham molecular orbitals representing the LE and CS states are shown in Figures S9–S11, SI.

A COSMO‐like model was applied to estimate the influence of a polar environment on the electronic excitations.^[22]^ Benzonitrile (BZN) was taken as the solvent. The ground state (GS) solvation energies of **NG‐1**⋅C_60_, **NG‐1**⋅(C_60_)_2_, and (**NG‐1**)_2_⋅C_60_ are found to be −0.35, −0.37, and −0.67 eV, respectively.

**Figure 5:**
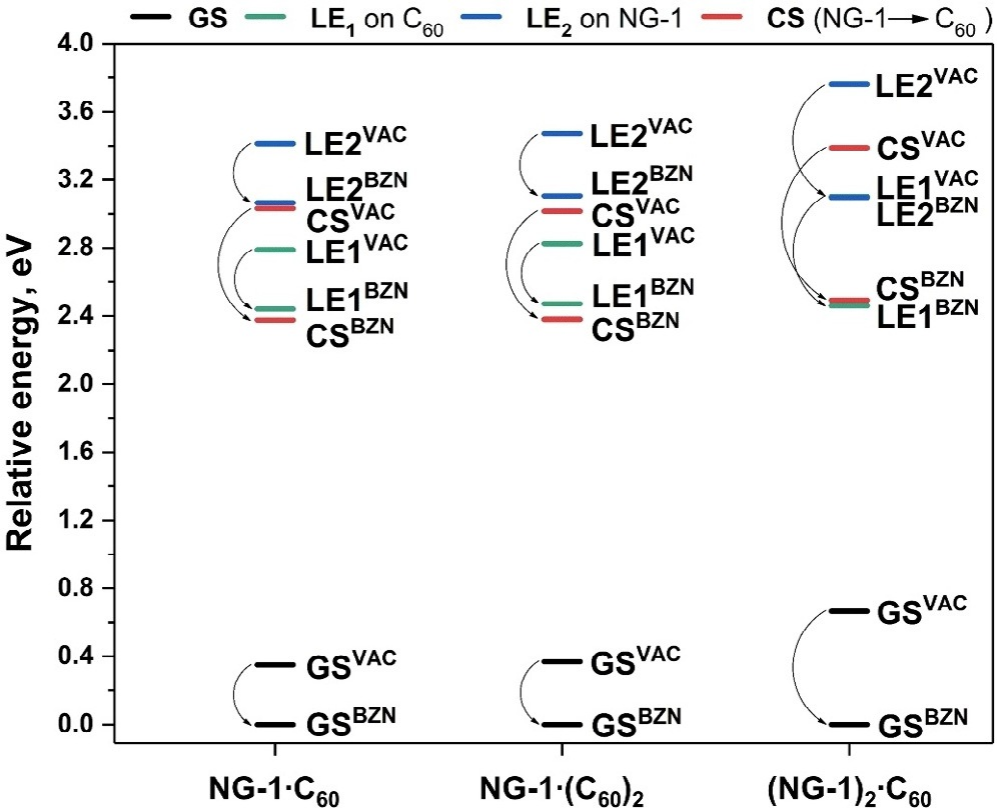
Relative energies of LE and CS states of **NG‐1**⋅**C_60_**, **NG‐1**⋅**(C_60_)_2_**, and **(NG‐1)_2_⋅C_60_** complexes computed in vacuum (VAC) and benzonitrile (BZN). The CS states correspond to electron transfer from **NG‐1** to C_60_ fragment.

As expected, the dipole moment and solvation energy of the LE_1_ and LE_2_ states are similar to those in the ground state (Table S6, SI). In contrast, the molecular dipole moment increases considerably as a result of the CS excitations. For **NG‐1**⋅C_60_, **NG‐1**⋅(C_60_)_2_, and (**NG‐1**)_2_⋅C_60_, the Δμ^CS^ is 22.5, 22.4, and 16.1 D, respectively. This in turn leads to a better stabilization of CS states by the polar environment as compared to the LE states (Figure 5). The calculations revealed that the CS state energy in the **NG‐1**⋅C_60_, **NG‐1**⋅(C_60_)_2_, and (**NG‐1**)_2_⋅C_60_ complexes decreases by 0.65, 0.63, and 0.89 eV, correspondingly. As seen in Figure 5, the solvent stabilization of the excited states makes the charge separation from LE_1_ energetically favorable. The LE state energies differ by less than 0.1 eV when compared to those of isolated C_60_ and **NG‐1** units (Table S7, SI).

The rate of electron transfer (*k*_ET_) was calculated using the Marcus equation.^[23]^ Within this approach, the rate is controlled by three parameters: electronic coupling *V*_ij_ of initial and final states, reorganization energy λ, and reaction Gibbs energy Δ*G* ^0^. Using the computed data listed in Table 1, we estimated the rates for the charge separation.

**Table 1:** Gibbs energy Δ*G* ^0^, electronic coupling |*V*_ij_|, reorganization energy λ, ET rate *k*_ET_, and characteristic time τ for charge separation in the **NG‐1⋅C_60_**, **NG‐1⋅(C_60_)_2_**, and **(NG‐1)_2_⋅C_60_** complexes in benzonitrile.

Complex	Transition	Δ*G* ^0[a]^ [eV]	|*V*_ij_| [eV]	Reorg. Energy [eV]	*k*_ET_ [s^−1^]	*τ* [ns]
**NG‐1⋅C_60_**	LE→CS	−0.063	6.01×10^−3^	0.545	1.30×10^10^	0.08
**NG‐1⋅(C_60_)_2_**	LE→CS	−0.091	4.35×10^−3^	0.440	1.98×10^10^	0.05
**(NG‐1)_2_⋅C_60_**	LE→CS	0.035	3.40×10^−3^	0.570	2.10×10^9^	0.48

[a] Gibbs energy difference between LE and CS states in benzonitrile.

The charge separation is characterized by a negative Δ*G* ^0^, except for (**NG‐1**)_2_⋅C_60_, where Δ*G* ^0^ is almost zero, and it occurs in the normal Marcus regime (|Δ*G* ^0^| < λ) on the picosecond time scale. Note that the charge transfer reaction in **NG‐1**⋅C_60_ and **NG‐1**⋅(C_60_)_2_ is significantly faster than in (**NG‐1**)_2_⋅C_60_.

To shed light onto the photo‐physical consequences that stem from the interaction between **NG‐1** and C_60_ we turned to steady‐state and time‐resolved investigations. The starting point were titrations, in which variable amounts of C_60_ up to 4.0×10^−5^ M were added to a constant concentration of **NG‐1** (2.0×10^−6^ M) and the absorption differences were subsequently recorded. No particular changes were, however, noted. As a matter of fact, the absorption spectra of **NG‐1** in the presence of C_60_ are best described as the simple superimposition of the individual constituents. An independent confirmation came from subtracting any contributions from C_60_ along every step of the titrations. Here, it is simply the unaltered **NG‐1** absorptions that are discernible. Quite similar is the outcome of the titrations when following the fluorescence rather than the absorption. In fact, the **NG‐1** fluorescence is subject to a minimal decrease, without showing any new fluorescent characteristics. Contributions from inner filter effects rendered it impossible to quantify the static interactions between **NG‐1** and C_60_. Considering the binding constants as they were determined in the NMR experiments (see above) complex formation under the conditions used in our spectroscopic experiments is disfavored.^[24]^

Information regarding the dynamic interactions, that is, energy versus electron transfer between **NG‐1** and C_60_, came from time‐resolved transient absorption measurements on the ns‐time scale (ns‐TAS). Initially, **NG‐1** was probed in 460 nm photo‐excitation experiments in the absence of any C_60_ (Figure S12, SI). The presence of two dominant species was derived from the ns‐TAS 3D heat maps. It is, on the one hand, the singlet excited state of **NG‐1** with a maximum at 665 nm. On the other hand, maxima at 550 and 690 nm as well as a minimum at 615 nm are ascribed to the triplet excited state of **NG‐1**.

All characteristics concluded from differential absorption spectra are in excellent agreement with the species associated spectra (SAS) obtained from Global Target Analysis. Our two‐species model is based on the lowest singlet and triplet excited states. Importantly, any branching coefficients were derived from FQY, while assuming that contributions from internal conversion are minor. All lifetimes are summarized in Table 2.

**Table 2:** Summary of the transient lifetimes determined by global target analysis for different molar ratios of **NG‐1** and C_60_.^[a]^

NG‐1⋅C_60_	1:0	1:5	1:10	1:15	1:25
Chlorobenzene					
S_1_ (NG‐1)	4.44 ns	4.44 ns	4.44 ns	–	4.44 ns
T_1_ (NG‐1)	22.3 μs	3.90 μs	2.07 μs	–	0.87 μs
T_1_ (C_60_)	–	26.8 μs	16.0 μs	–	31.4 μs
					
Benzonitrile					
S_1_ (NG‐1)	5.59 ns	5.59 ns	5.59 ns	5.59 ns	5.59 ns
T_1_ (NG‐1)	>100 μs	6.07 μs	4.26 μs	3.49 μs	2.69 μs
NG‐1^.+^ ‐C_60_^.−^	–	>100 μs	>100 μs	43.5 μs	22.9 μs

[a] In chlorobenzene and benzonitrile after laser excitation at 460 nm. The lifetime of the **NG‐1** singlet excited state was fixed during the global target analysis, since it only showed small deviations.

**Figure 6:**
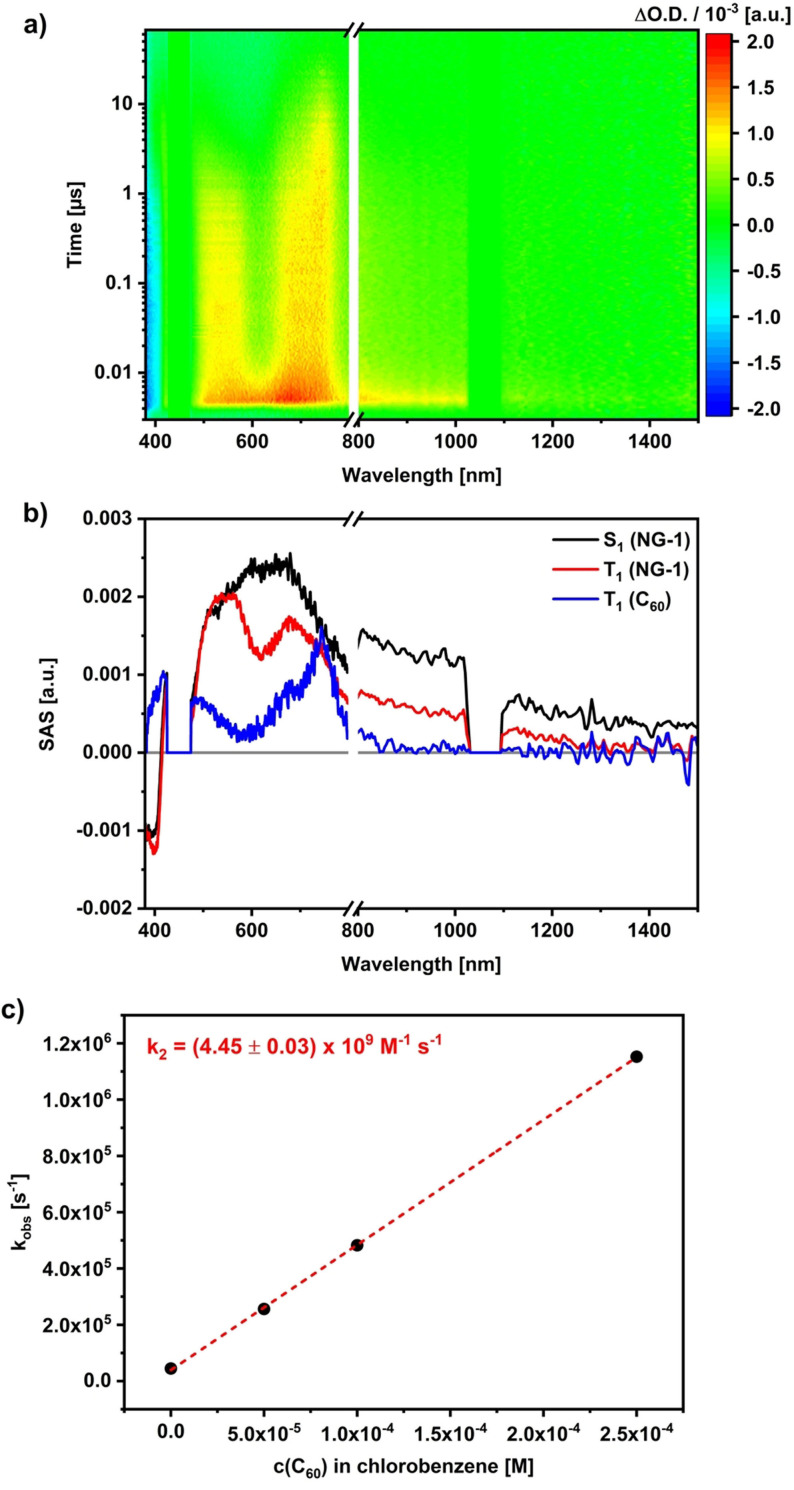
a) Differential transient absorption spectra of **NG‐1** (1.0×10^−5^ M) and C_60_ (1.0×10^−4^ M) in a 1:10 molar ratio at time delays between 0 and 70 μs after laser excitation at 460 nm in argon purged chlorobenzene. b) SAS obtained from global target analysis. c) Determination of the bimolecular rate constant *k*_2_ of the triplet excited state lifetime of **NG‐1** with increasing amount of C_60_.

**Figure 7:**
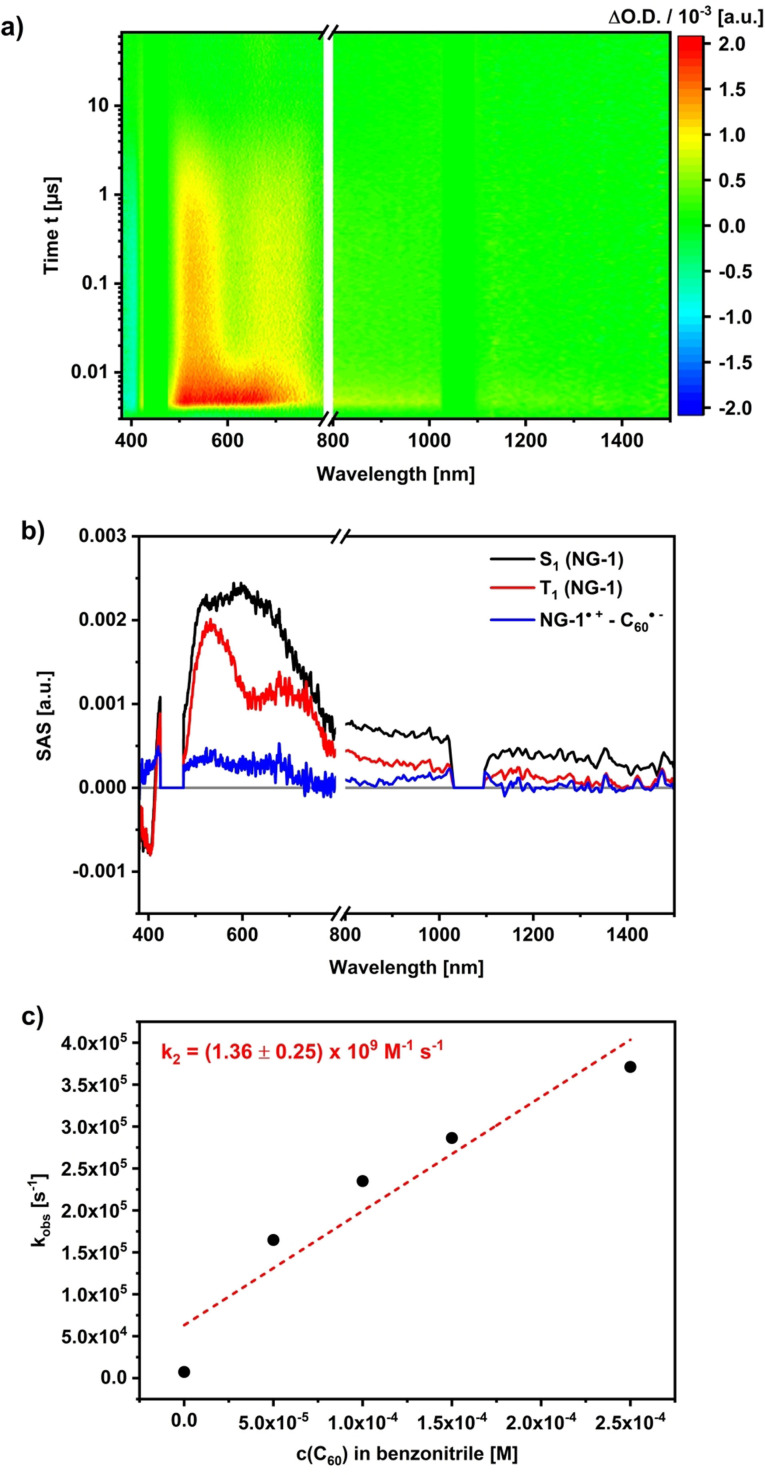
a) Differential transient absorption spectra of **NG‐1** (1.0×10^−5^ M) and C_60_ (1.0×10^−4^ M) in a 1:10 molar ratio at time delays between 0 and 70 μs after laser excitation at 460 nm in argon purged benzonitrile. b) SAS obtained from global target analysis. c) Determination of the bimolecular rate constant *k*_2_ of the triplet excited state lifetime of **NG‐1** with increasing amount of C_60_.

Adding C_60_ to **NG‐1** solutions to realize different **NG‐1**‐to‐C_60_ ratios of 1:5, 1:10 as well as 1:25 and photo‐exciting them at 460 nm led to some changes in the ns‐TAS 3D heat maps (Figure 6). Both the singlet and triplet excited states of **NG‐1** dominate the 3D heat maps, albeit with somewhat shorter lifetimes for the triplet excited state. As a major change, another species evolved on the long timescales, that is, >1 μs. It is linked to the faster decay of the **NG‐1** triplet excited state. Intriguingly, an analysis of the 3D heat maps reveals that the nature of this newly formed species is solvent dependent. In chlorobenzene, for example, the spectroscopic fingerprints of the C_60_ triplet excited state are seen to evolve at 750 nm.^[25]^ In benzonitrile, it is the one‐electron reduced form of C_60_ that is identified by its 1080 nm fingerprint (Figure 7).^[26]^ Global target analyses allowed deconvoluting the spectra for each component. Important is the fact that we took the ratio of the extinction coefficients of **NG‐1** and C_60_ at the excitation wavelength to account for the population of the C_60_ triplet excited state via ISC into our model. Such a mechanistic refinement helped in terms of the SAS quality of the **NG‐1** singlet and triplet excited states as the first and second species, respectively.^[27]^

The SAS of the third species bears in chlorobenzene the features of the C_60_ triplet excited state at 750 nm, while those of the C_60_ radical anion at 1080 nm were noted in benzonitrile. Using the pseudo first‐order rate constants and treating them as a function of C_60_ concentrations afforded second‐order rate constants for the energy transfer in chlorobenzene and the electron transfer in benzonitrile of 4.45×10^9^ and 1.36×10^9^ M^−1^ s^−1^, respectively. Both of them are nearly diffusion‐controlled.^[28]^

## Conclusion

The complexation between a corannulene‐based molecular nanographene **NG‐1** with both, negative and positive curvature and C_60_ was achieved. The ^1^H NMR titration performed by the addition of a solution of C_60_ to a solution of **NG‐1** showed the major shift of the protons close to the union between corannulene and the π‐extended system, where the curvature is the strongest. Concave‐convex interactions between the concave surface of the corannulene moiety of the molecular nanographene and C_60_ can result in the formation of the complex **NG‐1**⋅C_60_ with 1:1 stoichiometry and an association constant *K*_a_=1.17×10^3^ M^−1^. Furthermore, the saddle shape gathered by the negative curvature allows the formation of concave‐convex interactions with another molecule of C_60_ conducing to the formation of the complex **NG‐1**⋅(C_60_)_2_ with 1:2 stoichiometry and association constants *K*_a1_=1.69×10^3^ M^−1^ and *K*_a2_=1.16×10^3^ M^−1^. However, the best fit for the data extracted from the ^1^H NMR titration is for the complex (**NG‐1**)_2_⋅C_60_ with 2:1 stoichiometry where a C_60_ molecule remains encapsulated between the concave surface of the corannulene of two molecules of **NG‐1**. The measured association constants are *K*_a1_=1.71×10^3^ M^−1^ and *K*_a2_=3.17×10^3^ M^−1^.

Theoretical calculations of the stability of these complexes were performed computing the interaction energies (Δ*E*_int_) between **NG‐1** and C_60_. For the most stable conformers of **NG‐1**⋅C_60_, **NG‐1**⋅(C_60_)_2_, and (**NG‐1**)_2_⋅C_60_, Δ*E*_int_ is found to be −39.83, −66.98, and −84.94 kcal mol^−1^, respectively. Furthermore, population analysis lacked any evidence for a significant charge transfer between the fragments in the ground state. However, in the calculations of the excited states for the complexes with the three stoichiometries, we found: 1) locally excited (LE) states, in which the excitation is mainly localized on one fragment 2) Charge separated (CS) states with a significant amount of electron density transferred between the fragments and, 3) mixed states with a significant contribution of both LE and CS. The charge transfer reaction for **NG‐1**⋅C_60_ and **NG‐1**⋅(C_60_)_2_ is significantly faster than that for (**NG‐1**)_2_⋅C_60_.

Titration absorption and fluorescence studies do not show the formation of any complex, because the small association energies for the complexation reactions determined by NMR do not allow the complexation under the high dilution conditions of these spectroscopic methods. Time‐resolved transient absorption measurements on the ns‐time scale of solutions of **NG‐1** in the presence of C_60_ indicate solvent dependent, bimolecular processes. In chlorobenzene, it is triplet excited state energy transfer to yield the C_60_ triplet excited state. In polar benzonitrile, the detection of one‐electron reduced C_60_ confirms electron transfer.

This study validates the use of curved nanographenes as interesting and singular hosts for further concave‐convex supramolecular complexations and paves the way to the use of other nanographenes where shapes can play an essential role. Furthermore, molecular nanographenes exhibit appealing chemical, chiroptical and photophysical properties, which can be skillfully used in the formation of unprecedented non‐covalent complexes in the search for amazing controlled properties.

## Conflict of interest

The authors declare no conflict of interest.

## Supporting information

As a service to our authors and readers, this journal provides supporting information supplied by the authors. Such materials are peer reviewed and may be re‐organized for online delivery, but are not copy‐edited or typeset. Technical support issues arising from supporting information (other than missing files) should be addressed to the authors.

Supporting Information
