# Supplementary material for: Initiating Electron Transfer in Doubly Curved Nanographene Upon Supramolecular Complexation of C60
Source: Angew Chem Int Ed Engl. 2021 Dec 29;61(7):e202112834. doi: 10.1002/anie.202112834 (PMC9303211; doi:10.1002/anie.202112834)
Supplement: Supplementary file 1 — Supporting Information [file ANIE-61-0-s001.pdf]

Angewandte Chemie
Eine Zeitschrift der Gesellschaft Deutscher Chemiker

Supporting Information

Initiating Electron Transfer in Doubly Curved Nanographene Upon Supramolecular Complexation of C_{60}

Simon Zank[+], Jesús M. Fernández-García[+], Anton J. Stasyuk, Alexander A. Voityuk, Marcel Krug, Miquel Solà, Dirk M. Guldi,* and Nazario Martín**

anie_202112834_sm_miscellaneous_information.pdf

[+] These authors contributed equally to this work.

Content

1. General. ... 3

2. ^{1}H-NMR titration. ... 4

2.1. Procedure ... 4

2.2. Fitting models. ... 5

2.2.1 1:1 Stoichiometry ... 5

2.2.2. 1:2 Stoichiometry ... 6

2.2.3. 2:1 Stoichiometry ... 6

3. Theoretical calculations. ... 7

3.1 Methodology ... 7

3.1.1. Quantum-chemical calculations ... 7

3.1.2. Analysis of excited states ... 7

3.1.3 Solvent Effects 8
3.1.4. Electron transfer rates 8
3.2. Reorganization energy 9
3.3. Energy decomposition analysis 9
3.4. Non-covalent interactions (NCI) 9
4. Photophysical study. 23
4.1. Steady-state absorption and fluorescence spectroscopy 23
4.2. Transient absorption spectroscopy 23
5. References 27

1. General.

Unless otherwise noted, all materials including solvents were obtained from commercial suppliers and used without further purification. C_{60} 99.5 % was purchased from Solenne, toluene-d^8 99.6 % atom % D was purchased from Aldrich and **NG-1** was prepared according to the procedure reported in the literature.[1] ^{1}H NMR spectra of the titration were recorded at 300 (Bruker AVIII) MHz. ^{1}H NMR and 2D NMR spectra for the signals assignation were recorded at 500 (Bruker AV). Chemical shifts for ^{1}H NMR and ^{13}C NMR are expressed in parts per million (ppm) relative to the solvent. Data are reported as follows: chemical shift, multiplicity (s = singlet, bs = broad singlet, d = doublet), coupling constant (Hz), and integration.

2. ^{1}H-NMR titration.

2.1. Procedure

Complexation between curved **NG-1** as host (Table S1, yellow) and C_{60} as guest (Table S1, yellow) was performed by ^{1}H-NMR titration. In a NMR sample tube a $5x10^{-4}$ M solution of **NG-1** in toluene-d^8 was prepared. Sequential additions (4th column in Table S1) of a solution of C_{60} ($2x10^{-3}$ M) and **NG-1 (**$5x10^{-4}$ M, added to maintain the concentration of **NG-1** constant, table S1 3rd column) in toluene-d^8 were made. These additions led to the ^{1}H NMR spectra showed in figure S1 with chemical shifts values for protons H_a, H_b, H_c and H_d showed in green in table S1.

Table S1. Experimental data of ^{1}H-NMR titration of supramolecular complexation between **NG-1** and C_{60}.

Compounds		Vol (mL)	MW	Mass (mg)	Conc. M
Host	NG-1	2,5	975,33	1,219	5,00E-04
Guest	[60]fullerene	1,6	720,64	2,306	2,00E-03

	Solution of NG-1			Solution of C60					Total vol (mL)				
	Vol (mL)	n (mmol)	[NG-1] (M)	Vol added (mL)	Total vol added (mL)	n (mmol)	[C60]	Equiv C60		δ Ha	δ Hb	δ Hc	δ Hd
1	0,500	2,50E-04	5,00E-04	0,0000	0,0000	0,00E+00	0,00E+00	0,00	0,500	9,3570	9,2376	8,7651	7,9057
2	0,500	2,56E-04	5,00E-04	0,0125	0,0125	2,50E-05	4,88E-05	0,10	0,513	9,3551	9,2339	8,7620	7,9074
3	0,500	2,63E-04	5,00E-04	0,0125	0,0250	5,00E-05	9,52E-05	0,19	0,525	9,3570	9,2329	8,7570	7,9100
4	0,500	2,69E-04	5,00E-04	0,0125	0,0375	7,50E-05	1,40E-04	0,28	0,538	9,3588	9,2301	8,7549	7,9130
5	0,500	2,75E-04	5,00E-04	0,0125	0,0500	1,00E-04	1,82E-04	0,36	0,550	9,3592	9,2282	8,7520	7,9143
6	0,500	2,81E-04	5,00E-04	0,0125	0,0625	1,25E-04	2,22E-04	0,44	0,563	9,3601	9,2260	8,7496	7,9166
7	0,500	2,88E-04	5,00E-04	0,0125	0,0750	1,50E-04	2,61E-04	0,52	0,575	9,3630	9,2244	8,7467	7,9181
8	0,500	2,94E-04	5,00E-04	0,0125	0,0875	1,75E-04	2,98E-04	0,60	0,588	9,3613	9,2211	8,7432	7,9198
9	0,500	3,00E-04	5,00E-04	0,0125	0,1000	2,00E-04	3,33E-04	0,67	0,600	9,3636	9,2208	8,7450	7,9215
10	0,500	3,06E-04	5,00E-04	0,0125	0,1125	2,25E-04	3,67E-04	0,73	0,613	9,3632	9,2186	8,7403	7,9227
11	0,500	3,13E-04	5,00E-04	0,0125	0,1250	2,50E-04	4,00E-04	0,80	0,625	9,3648	9,2178	8,7395	7,9239
12	0,500	3,44E-04	5,00E-04	0,0625	0,1875	3,75E-04	5,45E-04	1,09	0,688	9,3644	9,2122	8,7323	7,9301
13	0,500	3,75E-04	5,00E-04	0,0625	0,2500	5,00E-04	6,67E-04	1,33	0,750	9,3680	9,2101	8,7269	7,9343
14	0,500	4,06E-04	5,00E-04	0,0625	0,3125	6,25E-04	7,69E-04	1,54	0,813	9,3695	9,2068	8,7261	7,9373
15	0,500	4,38E-04	5,00E-04	0,0625	0,3750	7,50E-04	8,57E-04	1,71	0,875	9,3712	9,2032	8,7206	7,9395
16	0,500	5,00E-04	5,00E-04	0,1250	0,5000	1,00E-03	1,00E-03	2,00	1,000	9,3708	9,2017	8,7165	7,9432
17	0,500	5,63E-04	5,00E-04	0,1250	0,6250	1,25E-03	1,11E-03	2,22	1,125	9,3746	9,1991	8,7130	7,9466
18	0,500	6,25E-04	5,00E-04	0,1250	0,7500	1,50E-03	1,20E-03	2,40	1,250	9,3747	9,1994	8,7120	7,9488
19	0,500	7,50E-04	5,00E-04	0,2500	1,0000	2,00E-03	1,33E-03	2,67	1,500	9,3746	9,1956	8,7097	7,9514
20	0,500	8,75E-04	5,00E-04	0,2500	1,2500	2,50E-03	1,43E-03	2,86	1,750	9,3768	9,1938	8,7081	7,9529

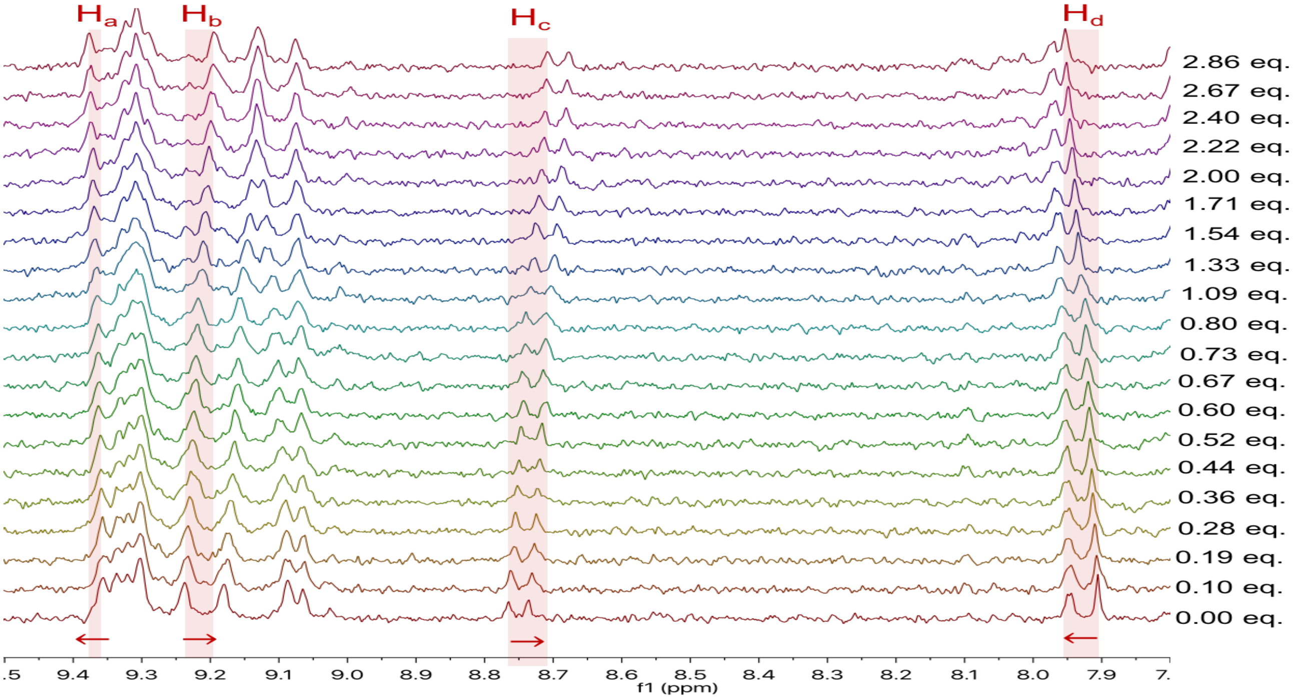

Figure S1. ^{1}H-NMR spectra resulting for the sequential additions of C_{60}.

2.2. Fitting models.

The calculation of association constants was made representing δ of each proton (Table S1 green columns) vs. $[G]_0/[H]_0$ (table S1 8th column) and fitting these points to the equations for changes of the ^{1}H NMR chemical shifts Δδ of the corresponding stoichiometric model.[2] The fit was performed by using Pall Thordarson's Bindfit open source software with L–BFGS–B method.[3]

2.2.1 1:1 Stoichiometry

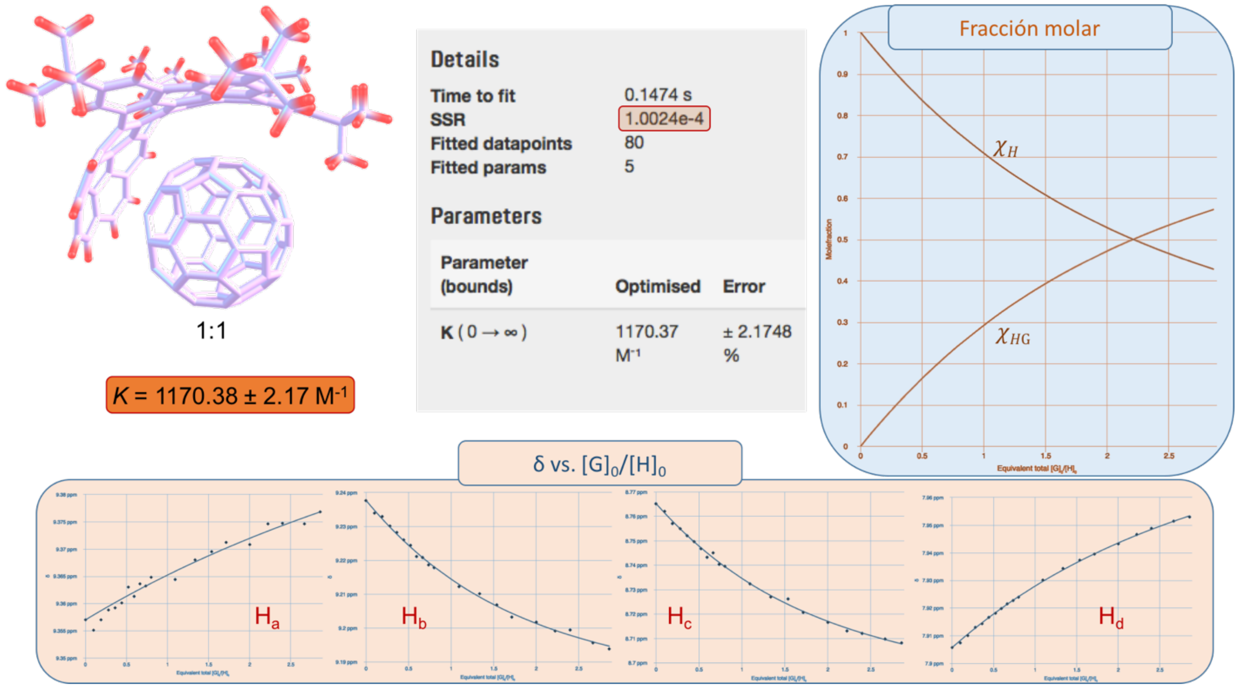

Figure S2. Association constants, mole fraction and fitting curves for 1:1 stoichiometry.

2.2.2. 1:2 Stoichiometry

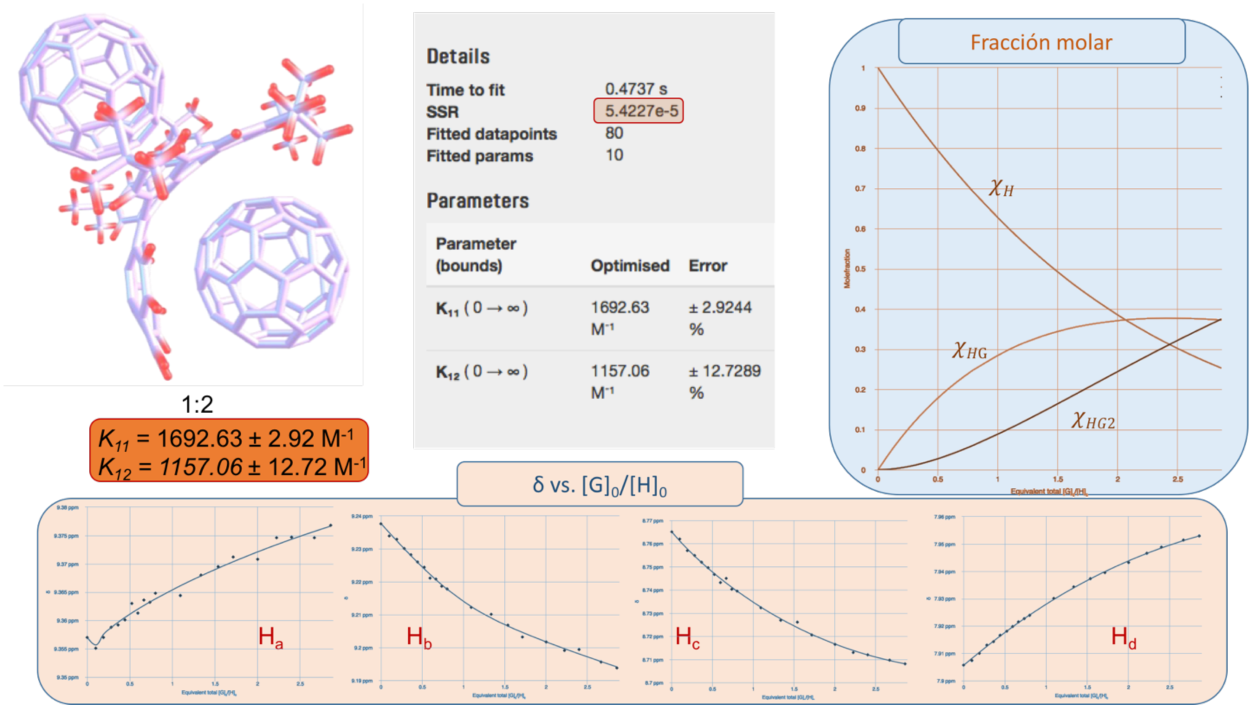

Figure S3. Association constants, mole fraction and fitting curves for 1:2 stoichiometry.

2.2.3. 2:1 Stoichiometry

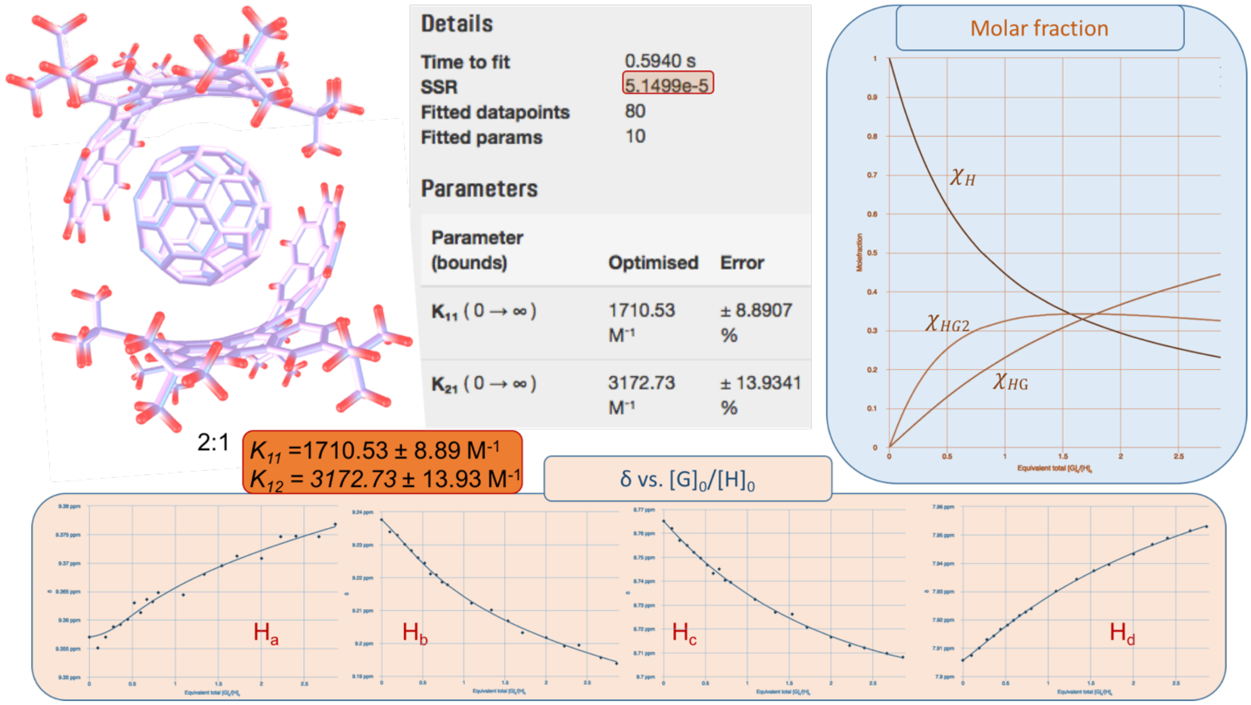

Figure S4. Association constants, mole fraction and fitting curves for 2:1 stoichiometry.

3. Theoretical calculations.

3.1 Methodology

3.1.1. Quantum-chemical calculations

Geometry optimization of the complexes was performed employing the DFT B3LYP[4,5] exchange−correlation functional with Ahlrichs' Def2-SVP basis set,[6,7] and using the resolution of identity approximation (RI, alternatively termed density fitting)[8,9] implemented in the ORCA 4.2.1 program.[10,11] The host-guest interaction energy was computed using B3LYP functional coupled with triple-ξ Def2-TZVP basis.[12] Vertical excitation energies were calculated using TDA formalism[13] with the range-separated functional from Handy and coworkers' CAM-B3LYP[14] and Ahlrichs' Def2-SVP basis set,[6,7] using Gaussian 16 (rev. A03).[15] The empirical dispersion D3 correction with Becke–Johnson damping was employed.[16,17] The population analysis performed within Mulliken,[18,19] Lowdin,[20] Hirshfeld,[21] and CM5[22] schemes were carried out using code implemented in Gaussian 16. The NCI method[23,24] was employed through the analysis of the reduced density gradient (RDG) at the CAM-B3LYP/Def2-SVP level using Multiwfn program.[25] To visualize molecular structures, NCI isosurfaces, and natural transition orbitals, the Chemcraft 1.8. program was used.[26]

3.1.2. Analysis of excited states

The quantitative analysis of exciton delocalization and charge transfer in the donor-acceptor complexes is carried out in terms of transition density.[27-29] The analysis is convenient to perform in the Löwdin orthogonalized basis. The matrix $^{\lambda}\mathbf{C}$ of orthogonalized MO coefficients is obtained from the coefficients $\mathbf{C}$ in the original basis $^{\lambda}\mathbf{C} = \mathbf{S}^{1/2}\,\mathbf{C}$, where $\mathbf{S}$ is the atomic orbital overlap matrix. The transition density matrix T^{0i} for an excited state Φ^* constructed as a superposition of singly excited configurations (where an occupied MO ψ_i is replaced a virtual MO ψ_a) is computed

$$T^{0i}_{\alpha\beta} = \sum_{ia} A_{i\to a}\,{}^{\lambda}C_{\alpha i}\,{}^{\lambda}C_{\beta a} \quad , \qquad (1)$$

where $A_{i\to a}$ is the expansion coefficient and α and β are atomic orbitals.

A key quantity Ω (D,A) is determined by

$$\Omega(D,A) = \sum_{\alpha\in D,\,\beta\in A} \left(T^{0i}_{\alpha\beta}\right)^2 \qquad (2)$$

The weights of local excitations on D and A are Ω(D,D) and Ω(A,A). The weight of electron transfer configurations D→A and A→D is represented by Ω(D,A) and Ω(A,D), respectively. The index Δq, which describes charge separation and charge transfer between D and A, is

$$\Delta q(CS) = \sum \Omega(D,A) - \Omega(A,D) \qquad (3)$$

$$\Delta q(CT) = \sum \Omega(D,A) + \Omega(A,D) \qquad (4)$$

3.1.3 Solvent Effects

The equilibrium solvation energy E_S^{eq} of a molecule (in the ground or excited state) in the medium with the dielectric constant ε was estimated using a COSMO-like polarizable continuum model[30,31] in monopole approximation:

$$E_S^{eq}(Q,\varepsilon) = -\frac{1}{2} f(\varepsilon) Q^{+} D Q \tag{5}$$

where the $f(\varepsilon)$ is the dielectric scaling factor, $f(\varepsilon) = \frac{\varepsilon - 1}{\varepsilon}$, **Q** is the vector of *n* atomic charges in the molecular system, and **D** is the *n* x *n* symmetric matrix determined by the shape of the boundary surface between solute and solvent. $\mathbf{D}=\mathbf{B}^{+}\mathbf{A}^{-1}\mathbf{B}$, where the *m* x *m* matrix **A** describes electrostatic interaction between *m* surface charges and the *m* x *n* **B** matrix describes the interaction of the surface charges with *n* atomic charges of the solute.[32-34] The GEPOL93 scheme[35] was used to construct the molecular boundary surface.

The charge on atom X in the excited state Φ_i, q_X^i, was calculated as:

$$q_X^i = q_X^0 + \Delta_X^i, \quad \Delta_X^i = \sum_{Y \neq X} \sum_{\alpha \in X, \beta \in Y} (T_{\alpha\beta}^{0i} T_{\alpha\beta}^{0i} - T_{\beta\alpha}^{0i} T_{\beta\alpha}^{0i}), \tag{6}$$

where q_X^0 is the atomic charge on X in the ground state and Δ_X^i is its change due to the redistribution of the electron density between the atoms X and Y which is caused by the excitation $\psi_0 \rightarrow \psi_i$.

The non-equilibrium solvation energy for excited state ψ_i can be estimated as:[36]

$$E_S^{neq}(Q^0, \Delta, \varepsilon, n^2) = f(\varepsilon) \Delta^{+} D Q^0 - \frac{1}{2} f(n^2) \Delta^{+} D \Delta, \tag{7}$$

In Eq. (7), n^2 (the refraction index squared) is the optical dielectric constant of the medium and the vector Δ describes the change of atomic charges in the molecule by excitation in terms of atomic charges, see Eq. (6). By definition, the external (solvent) reorganization energy is the difference of the non-equilibrium (Eq. 7) and equilibrium solvation (Eq. 5) energies of the excited state.

3.1.4. Electron transfer rates

The rate of the nonadiabatic ET, k_{ET}, can be expressed in terms of the electronic coupling squared, V^2, and the Franck-Condon Weighted Density of states (FCWD):

$$k_{ET} = \frac{2\pi}{} V^2 \left(FCWD\right) \tag{8}$$

that accounts for the overlap of vibrational states of donor and acceptor and can be approximately estimated using the classical Marcus equation:[37]

$$\left(FCWD\right) = \left(4\pi\lambda kT\right)^{-1/2} \exp\left[-\left(\Delta G^0 + \lambda\right)^2 / 4\lambda kT\right] \tag{9}$$

where λ is the reorganization energy and ΔG^0 is the standard Gibbs energy change of the process. The fragment charge difference (FCD)[38,39] method was employed to calculate the electronic couplings in this work.

3.2. Reorganization energy

The reorganization energy is usually divided into two parts, $\lambda = \lambda_i + \lambda_s$, including the internal and solvent terms. Solvent reorganization energy corresponds to the energy required to move solvent molecules from the position they occupy in the initial ground state (GS) to the location they have in the CT state, but without charge transfer having occurred. The λ_s for particular CT states were computed as a difference between equilibrium and non-equilibrium solvation energies. The internal reorganization energy λ_i corresponds to the energy of structural change when denoted fragments going from initial-state geometries to final-state geometries.

3.3. Energy decomposition analysis

The interaction energy in the gas phase is examined in the framework of the Kohn-Sham MO model using a quantitative energy decomposition analysis (EDA)[40-42] into electrostatic interactions, Pauli repulsive orbital interactions, and attractive orbital interactions, to which a term ΔE_{disp} is added to account for the dispersion correction:

$$\Delta E_{\mathrm{int}} = \Delta E_{elstat} + \Delta E_{Pauli} + \Delta E_{oi} + \Delta E_{disp} \tag{10}$$

The term $\Delta V_{\mathrm{elstat}}$ corresponds to the classical electrostatic interactions between the unperturbed charge distributions of the prepared (i.e. deformed) bases and is usually attractive. The Pauli repulsion, $\Delta E_{\mathrm{Pauli}}$, comprises the destabilizing interactions between occupied orbitals and is responsible for any steric repulsion. The orbital interaction, ΔE_{oi}, accounts for electron-pair bonding, charge transfer (*i.e.*, donor–acceptor interactions between occupied orbitals on one moiety and unoccupied orbitals on the other, including the HOMO-LUMO interactions) and polarization (empty-occupied orbital mixing on one fragment due to the presence of another fragment). The term ΔE_{disp} accounts for the dispersion corrections.[43,44]

3.4. Non-covalent interactions (NCI)

The NCI method[23,24] relies on two scalar fields to map local bonding properties: the electron density (ρ) and the reduced-density gradient (RDG, s), defined as:

$$s = \frac{1}{2(3\pi)^{1/3}} \frac{|\nabla\rho|}{\rho^{4/3}} \tag{11}$$

a quantity that is essential to the design of DFT functionals. The combination of s and ρ allows a rough partition of real space into bonding regions: high-s low-ρ corresponds to non-interacting density tails, low-s high-ρ to covalent bonds, and low-s low-ρ to non-covalent interactions.

Table S2. Comparison of the interaction energies (ΔE^{Tot}_{int}, kcal/mol) between **NG-1** and C_{60} in **NG-1**•C_{60} estimated with BLYP-D3(BJ)/def2-TZVP//BLYP-D3(BJ)/def2-SVP level of theory.

**Complex**	**Functional**			
	**BLYP**	**BLYP+BSSE**	**B3LYP**	**CAM-B3LYP**
**NG-1**•C_{60}	-39.87	-37.93	-35.63	-30.78
**NG-1**•$(C_{60})_2$	-66.98	n/a	-59.71	-51.68
(**NG-1**)$_2$•C_{60}	-84.94	n/a	-75.46	-64.15

$$\Delta E_{int}^{Tot} = E^{Complex} - \sum_{i=1}^{n} E_i^{Fragment}, where\ n = 2\ or\ 3$$

Table S3. Charge separation (units are electrons) between the fragments in electronic ground state for **NG-1·C_{60}, NG-1·$(C_{60})_2$,** and **$(NG-1)_2$·C_{60}**. Q_{NG-1} - charge on **NG-1**, and $Q_{C_{60}}$ - charge on fullerene moiety. The total charge of the complex is zero in all cases.

Charge	**NG-1·C_{60}**		**NG-1·$(C_{60})_2$**			**$(NG-1)_2$·C_{60}**		
	Q_{NG-1}	$Q_{C_{60}}$	Q_{NG-1}	$Q_{C_{60}^{1}}$	$Q_{C_{60}^{2}}$	$Q_{NG-1^{1}}$	$Q_{NG-1^{2}}$	$Q_{C_{60}}$
Mulliken	0.039	-0.039	0.050	-0.025	-0.025	0.011	0.027	-0.039
Löwdin	0.044	-0.044	0.067	-0.033	-0.033	0.026	0.032	-0.058
Hirshfeld	-0.006	0.006	-0.044	0.028	0.016	-0.045	-0.035	0.081
CM5	-0.001	0.001	-0.034	0.022	0.013	-0.040	-0.030	0.070

Table S4. EDA results for **NG-1·C_{60}, NG-1·(C_{60})$_2$,** and **(NG-1)$_2$·C_{60}** computed at the BLYP-D3(BJ)/def2-TZVP//BLYP-D3(BJ)/def2-SVP level of theory.[a]

Complex	Fragments		Energy terms				
	F1	F2	ΔE_{Pauli}	ΔE_{elstat}	ΔE_{oi}	ΔE_{disp}	ΔE_{int}
**NG-1·C_{60}**	NG-1	C_{60}	73.24	-32.23 (28.5%)	-15.24 (13.5%)	-65.60 (58.0%)	-36.67
**NG-1·(C_{60})$_2$**	NG-1·C_{60}	C_{60}	53.78	-22.20 (26.0%)	-12.51 (14.6%)	-50.81 (59.4%)	-31.74
**(NG-1)$_2$·C_{60}**	NG-1·C_{60}	NG-1	82.60	-33.05 (22.9%)	-17.33 (13.4%)	-78.27 (60.7%)	-46.31

[a] The energy values are in kcal/mol. The percentage contributions to the sum of all attractive energy terms are given in parentheses.

Table S5. Excitation energies (E_x, eV), main singly excited configuration (HOMO(H)–LUMO(L)) and its weight (W), oscillator strength (f), extent of charge separation (CS, e) or localization of exciton (X) in the **NG-1·C_{60}, NG-1·(C_{60})$_2$,** and **(NG-1)$_2$·C_{60}**.

	**Supramolecular assemblies**					
	**NG-1·C_{60}**		**NG-1·(C_{60})$_2$**		**(NG-1)$_2$·C_{60}**	
	VAC	BZN	VAC	BZN	VAC	BZN
	LE1 (on **C_{60}**)					
E_x	2.487	2.440	2.457	2.472	2.482	2.459
Transition (W)	H-3 – L+1 (0.20)	H-3 – L+1 (0.21)	H-3 – L+5 (0.46)	H-3 – L+5 (0.44)	H-7 – L (0.15)	H-8 – L (0.18)
F	<0.001	<0.001	<0.001	<0.001	<0.001	<0.001
X	0.878	0.923	0.885	0.913	0.889	0.891
	LE2 on (on **NG-1**)					
E_x	3.064	3.066	3.102	3.106	3.096	3.096
Transition (W)	H – L+3 (0.60)	H-1 – L+3 (0.53)	H – L+6 (0.48)	H – L+6 (0.68)	H – L+4 (0.41)	H – L+4 (0.52)
f	0.160	0.280	0.080	0.211	0.117	0.244
X	0.865	0.840	0.848	0.840	0.761	0.855
	Most absorptive transition					
E_x	3.833*	3.667*	3.867*	3.737*	3.889*	3.871*
Transition (W)	H-8 – L+3 (0.13)	H-1 – L+8 (0.09)	H-4 – L+4 (0.11)	H-2 – L+5 (0.13)	H-2 – L+5 (0.10)	H-4 – L+3 (0.10)
f	0.316	0.771	0.416	0.820	0.736	0.851
Localization	**NG-1**	**NG-1**	**NG-1**	**NG-1**	**NG-1**	**NG-1**
X	0.573	0.690	0.593	0.630	0.797	0.644
CT	0.346	0.291	0.216	0.305	0.176	0.277
	CS (**NG-1** → **C_{60}**)					
E_x	2.681	2.377	2.646	2.381	2.724	2.494

Transition (W)	H – L (0.33)	H – L+2 (0.38)	H – L+5 (0.57)	H – L+5 (0.65)	H-1 – L (0.60)	H-1 – L (0.69)
f	< 0.001	< 0.001	< 0.001	0.005	0.007	0.008
CT	0.853	0.800	0.810	0.879	0.880	0.881

* - mixed states with significant contributions of both LE and CS.

Table S6. Excitation energies (E_x, eV) and dipole moments in ground state (μ_0, D), change in dipole moments between ground state and state of interest ($\Delta\mu = \mu_i - \mu_0$, D), and solvation energies (E_{solv}, eV) for the studied supramolecular assemblies calculated in DCM.

	**Supramolecular host-guest systems**		
	**NG-1·C_{60}**	**NG-1·$(C_{60})_2$**	**$(NG\text{-}1)_2$·C_{60}**
	Ground state (GS)		
E_x	0.000	0.000	0.000
μ_0	3.06	2.76	0.55
E_{solv}	-0.350	-0.369	-0.665
	LE1 (on **C_{60}**)		
E_x	2.440	2.472	2.459
$\Delta\mu$	4.83	2.85	1.15
E_{solv}	-0.349	-0.354	-0.645
	LE2 on (on **NG-1**)		
E_x	3.066	3.106	3.096
$\Delta\mu$	6.36	3.77	6.86
E_{solv}	-0.348	-0.365	-0.665
	CS (**NG-1** → **C_{60}**)		
E_x	2.377	2.381	2.494
$\Delta\mu$	22.48	22.43	16.08
E_{solv}	-0.654	-0.634	-0.895

In order to estimate the effect of complexation on the excited state properties, we have compared the lowest-lying LE states of the studied complexes with the excited states of independent **C_{60}** and **NG-1** units. As seen from Table S7, the energetics of both LE types in the complexes is very similar to the lowest excited states energy in the corresponding monomers. The difference in energy has been found to be less than 0.1 eV. For all complexes, the excited state with maximum absorption is localized on the **NG-1** fragment and lies at 3.8-3.9 eV. Despite the fact that in isolated **NG-1** the highly absorptive state has a similar energy, it is characterized by a significantly stronger oscillator strength. The observed decrease in the oscillator strength can be explained by a change in the nature of such state. In all complexes, the excited state with high absorption is the mixed states with significant contributions of both LE and CT, while in isolated **NG-1** no charge transfer processes are possible and these stats is purely LE. The greater contribution of CT, the weaker oscillator strength. Therefore, in isolated **NG-1** the oscillator strength has the highest value.

Table S7. Excitation energies (E_x, eV), oscillator strength (f), and extent of charge separation (CS, e) for LE1, LE2 and most absorbtive exited states in **NG-1·C_{60}, NG-1·$(C_{60})_2$,** and **$(NG-1)_2$·C_{60}** systems in comparison with **C_{60}** and **NG-1** monomers.

		System				
		**NG-1·C_{60}**	**NG-1·$(C_{60})_2$**	**$(NG-1)_2$·C_{60}**	**C_{60}**	**NG-1**
LE1 on **C_{60}**						
	E_x	2.487	2.457	2.482	2.523	n/a
	f	<0.001	<0.001	<0.001	<0.001	
LE2 on **NG-1**						
	E_x	3.064	3.102	3.096	n/a	3.081
	f	0.160	0.080	0.117		0.169
Most Absorptive on NG-1						
	E_x	3.833*	3.867*	3.889*	n/a	3.941
	f	0.316	0.416	0.736		1.498
	CS	0.346	0.216	0.176		0.00

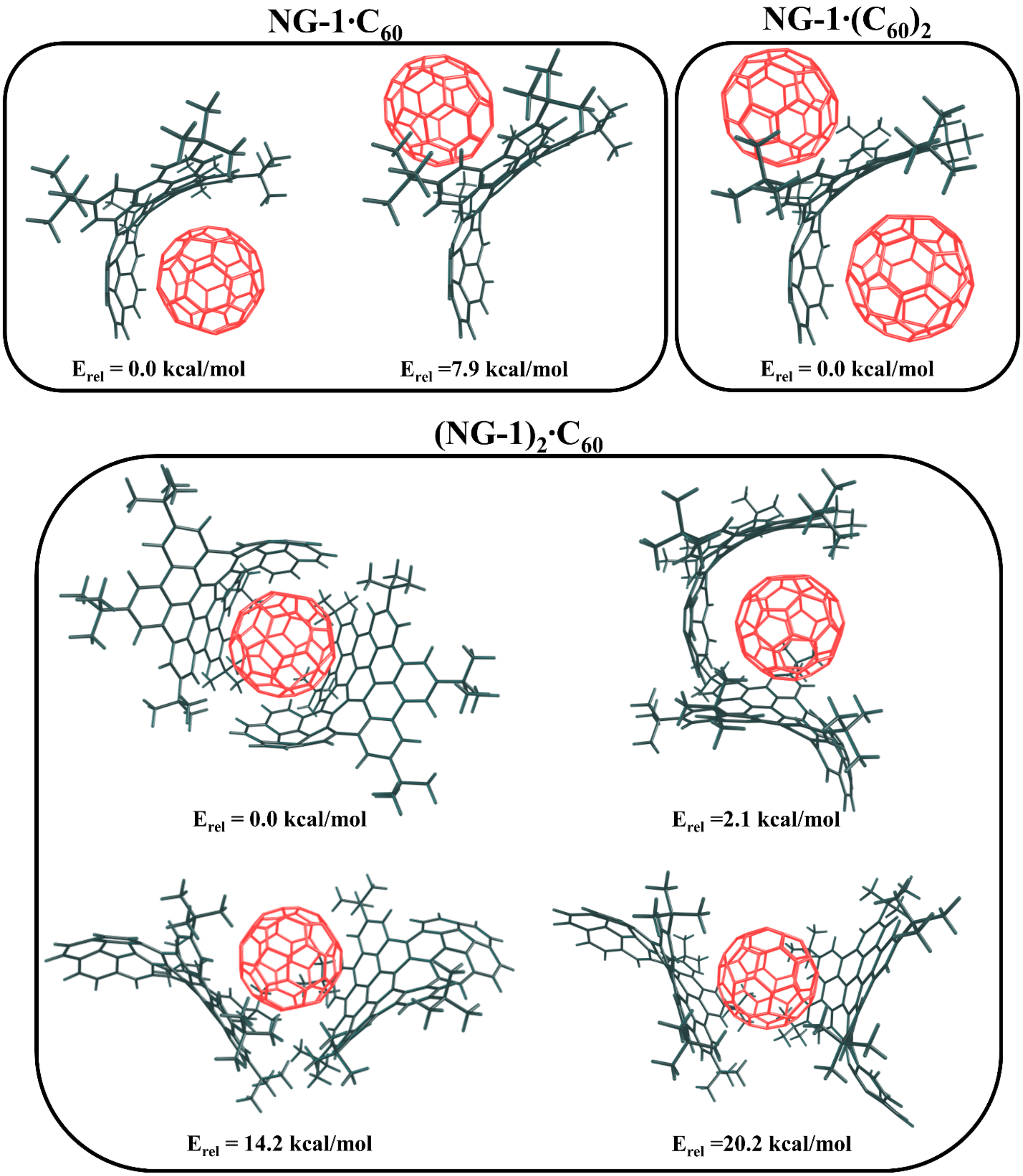

Figure S5. Structures of the found isomers for **NG-1·C_{60}, NG-1·$(C_{60})_2$,** and **$(NG\text{-}1)_2$·C_{60}** and their corresponding relative energies obtained at the BLYP-D3(BJ)/def2-SVP level of theory.

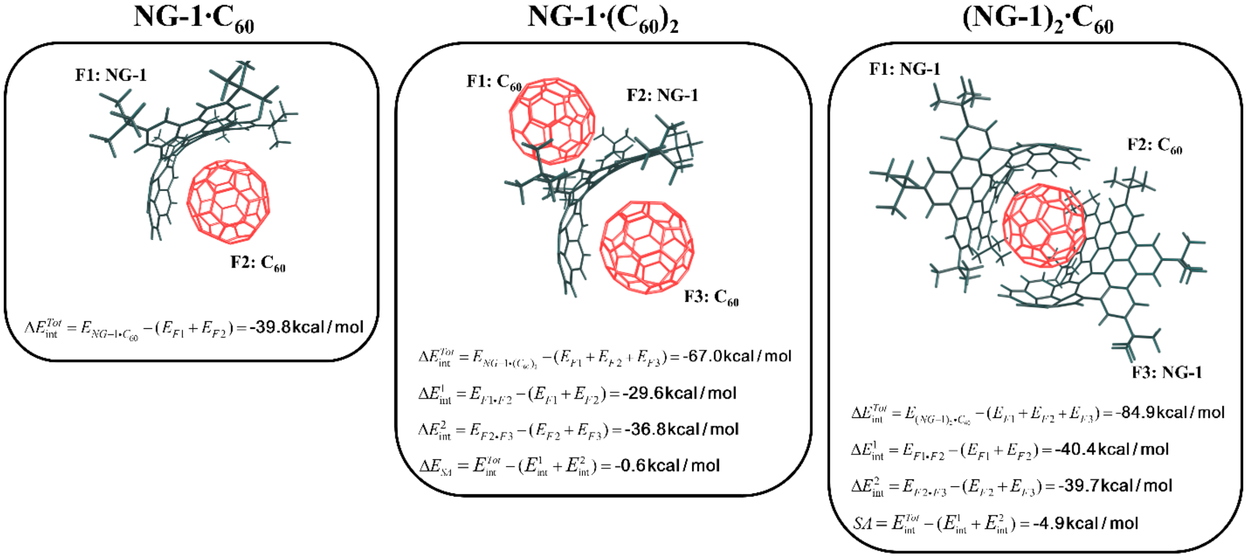

Figure S6. The interaction energies (ΔE_{int}, kcal/mol) between fragments and superadditivity values (ΔE_{SA}, kcal/mol) for **NG-1·C_{60}**, **NG-1·$(C_{60})_2$**, and **$(NG-1)_2$·C_{60}** obtained at the BLYP-D3(BJ)/def2-TZVP//BLYP-D3(BJ)/def2-SVP level of theory.

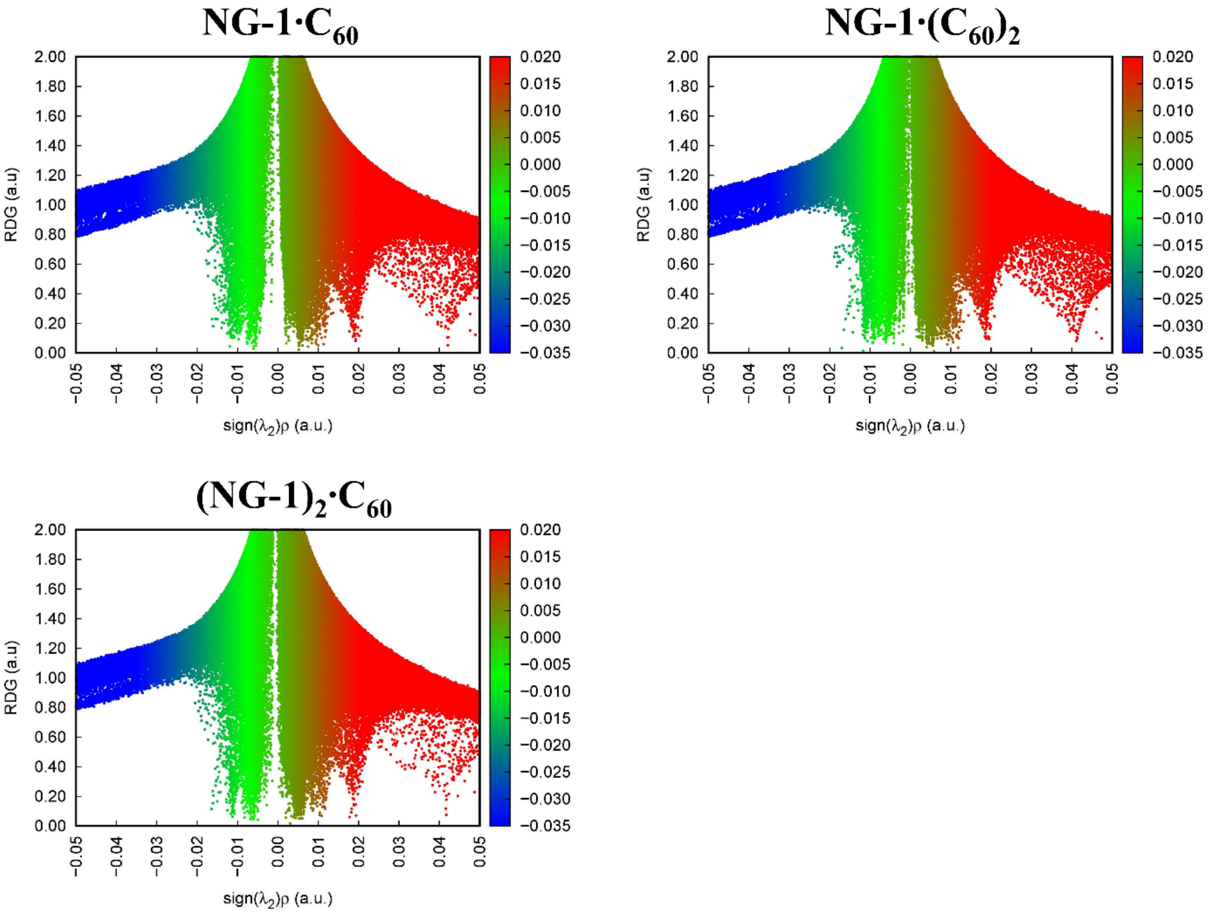

Figure S7. NCI Plot RDG *vs.* sign(λ2)×ρ for $\mathbf{NG\text{-}1{\cdot}C_{60}}$, $\mathbf{NG\text{-}1{\cdot}(C_{60})_2}$, and $\mathbf{(NG\text{-}1)_2{\cdot}C_{60}}$.

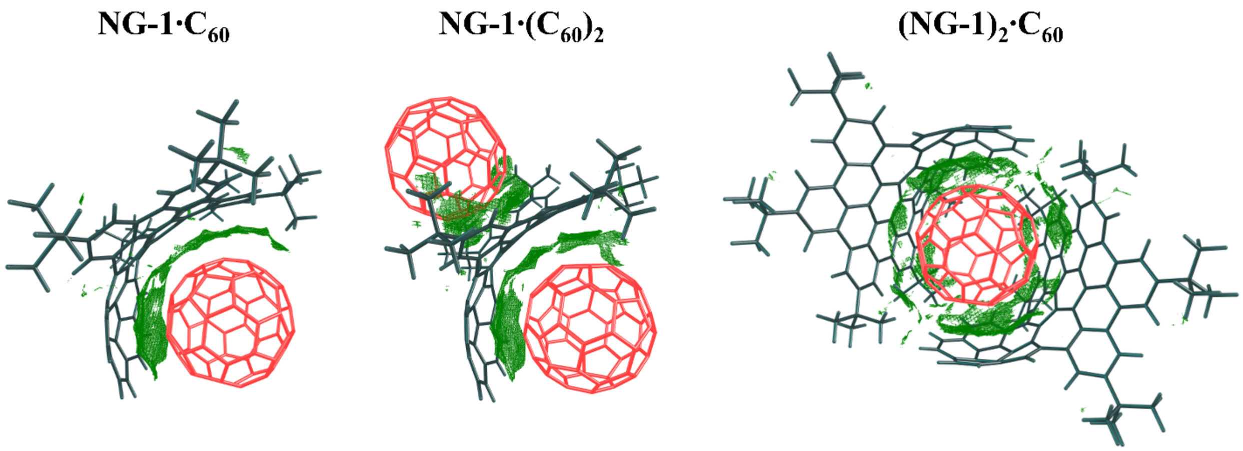

Figure 8. NCI isosurfaces of van der Waals interactions ($-0.008 < \mathrm{sign}(\lambda 2)\times\rho < 0.008$ a.u.) for **NG-1·C_{60}**, **NG-1·$(C_{60})_2$**, and **$(NG\text{-}1)_2$·C_{60}**. Isosurfaces were generated for RDG = 0.6 a.u.

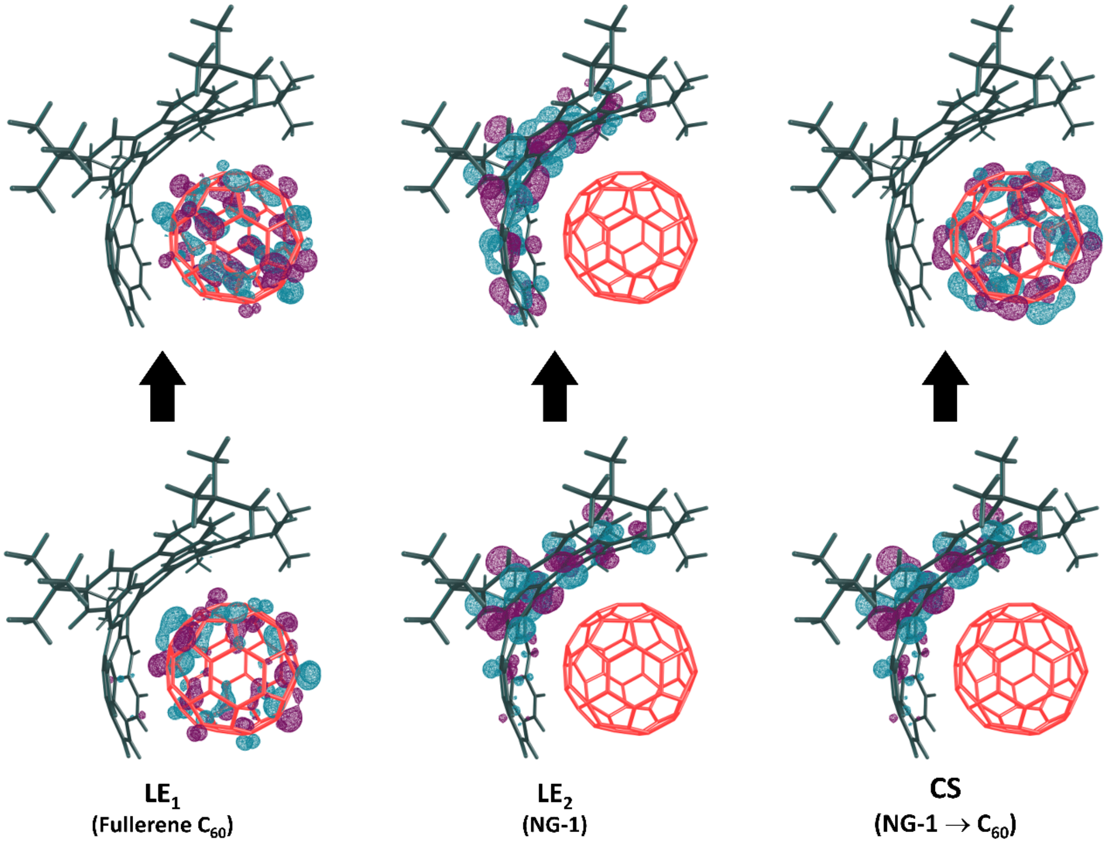

Figure 9. Kohn-Sham molecular orbitals representing the LE1, LE2, and CS states for **NG-1·C_{60}**. Isosurfaces were generated for 0.30 au.

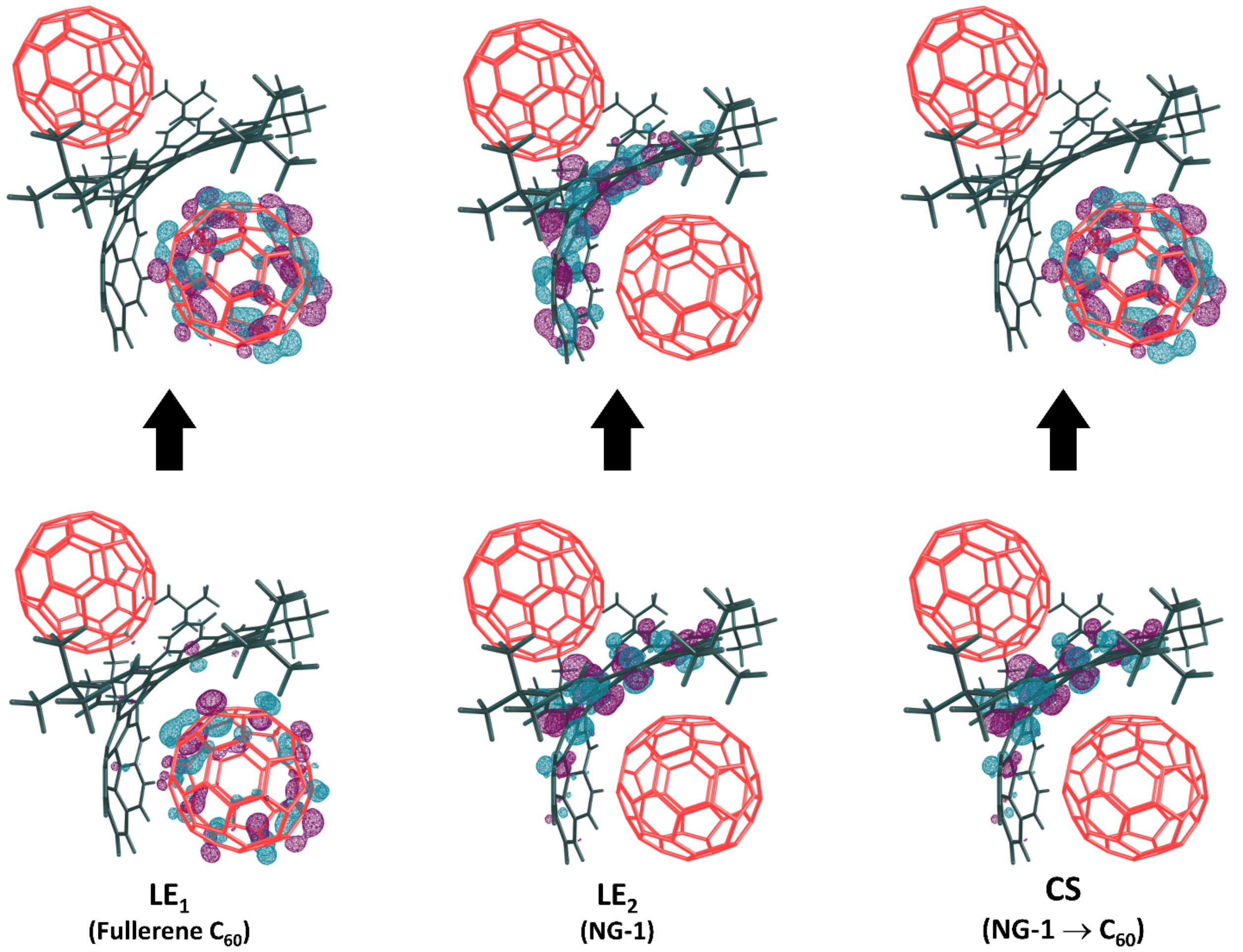

Figure 10. Kohn-Sham molecular orbitals representing the LE1, LE2, and CS states for **NG-1·(C_{60})$_2$**. Isosurfaces were generated for 0.30 au.

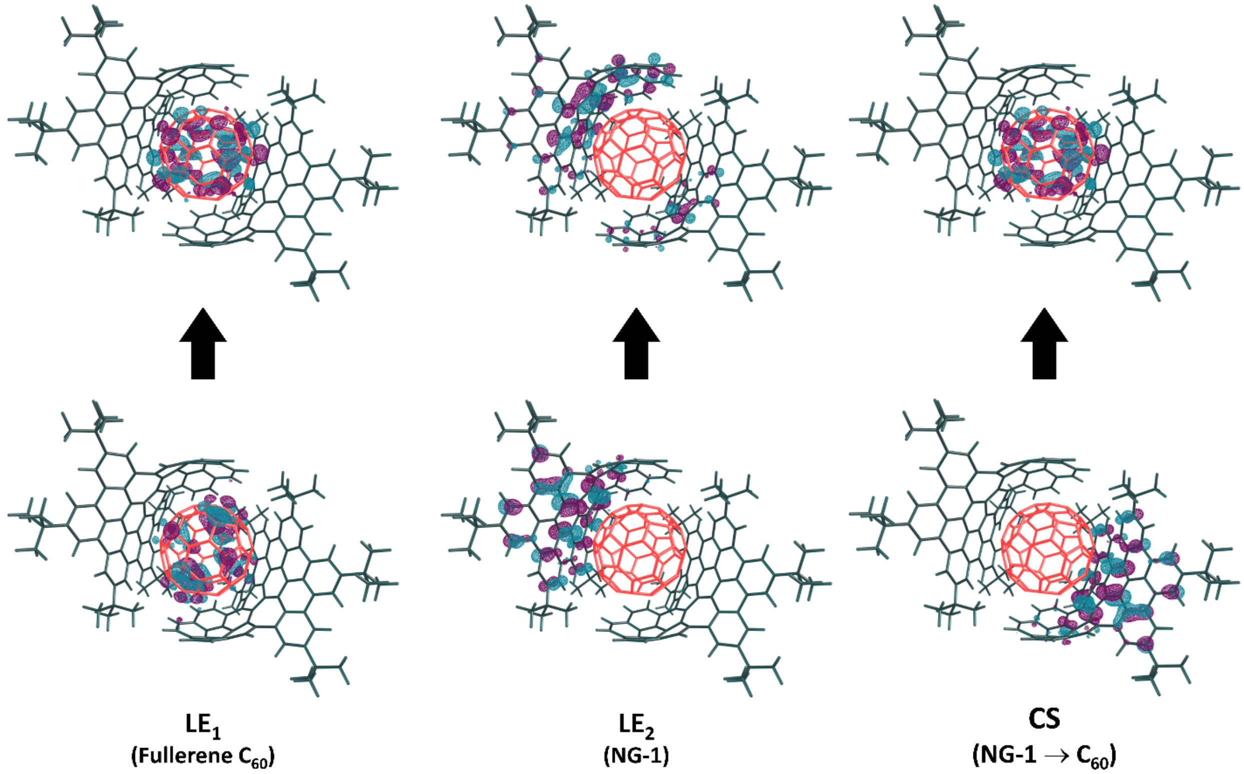

Figure 11. Kohn-Sham molecular orbitals representing the LE1, LE2, and CS states for **$(NG\text{-}1)_2{\cdot}C_{60}$**. Isosurfaces were generated for 0.30 au.

4. Photophysical study.

4.1. Steady-state absorption and fluorescence spectroscopy

Steady-state absorption spectroscopy was performed using a Perkin-Elmer Lambda 2 dual-beam spectrometer with a beam split of 2 nm and a scan rate of 480 nm/s. Fluorescence spectra were recorded on a Yobin FluoroMax 3 spectrometer with excitation and emission slit widths of 2 nm and an integration time of 0.25 s. Solutions were measured in 10 x 10 mm quartz cuvettes.

4.2. Transient absorption spectroscopy

Nanosecond transient absorption spectroscopy experiments were performed with the EOS detection system from Ultrafast Systems. A CPA-2101 laser from Clark-MXR with a Ti:sapphire crystal was used to generate the initial wavelength of 775 nm at a frequency of 1 kHz and 150 fs pulse width. Further, the excitation wavelength was tuned to 460 nm by non-linear optical parametric amplification (NOPA). A second laser generated the white light probe pulse. Light was detected using two CCD cameras, one recording in the UV-Vis and the other in the nIR range. Samples were prepared in 2 mm quartz cuvettes, degassed with argon for 15 min and stirred during the measurements.

Global target analysis was carried out using the Glotaran software with the R-package TIMP.[45]

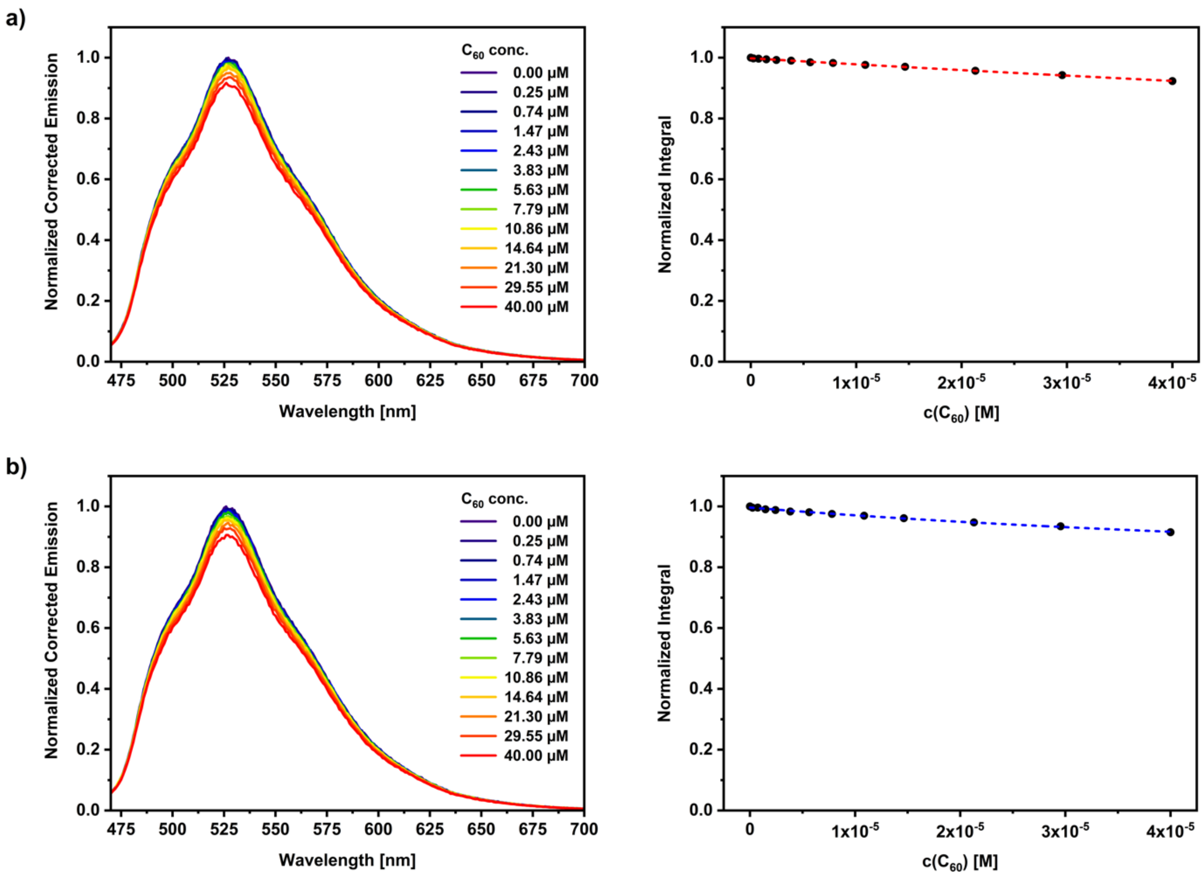

Figure S12. Normalized fluorescence spectra, which were obtained during the titration of **NG-1** with C_{60} in chlorobenzene. Fits for the binding constant are shown on the right. The titration was performed twice to confirm repeatability, so both titrations a) and b) follow the exact same procedure. Excitation was at 460 nm and concentration of **NG-1** was held constant during the measurements (2.0 x 10^{-6} M). The spectra were corrected with reference solutions containing only C_{60} at the same concentration.

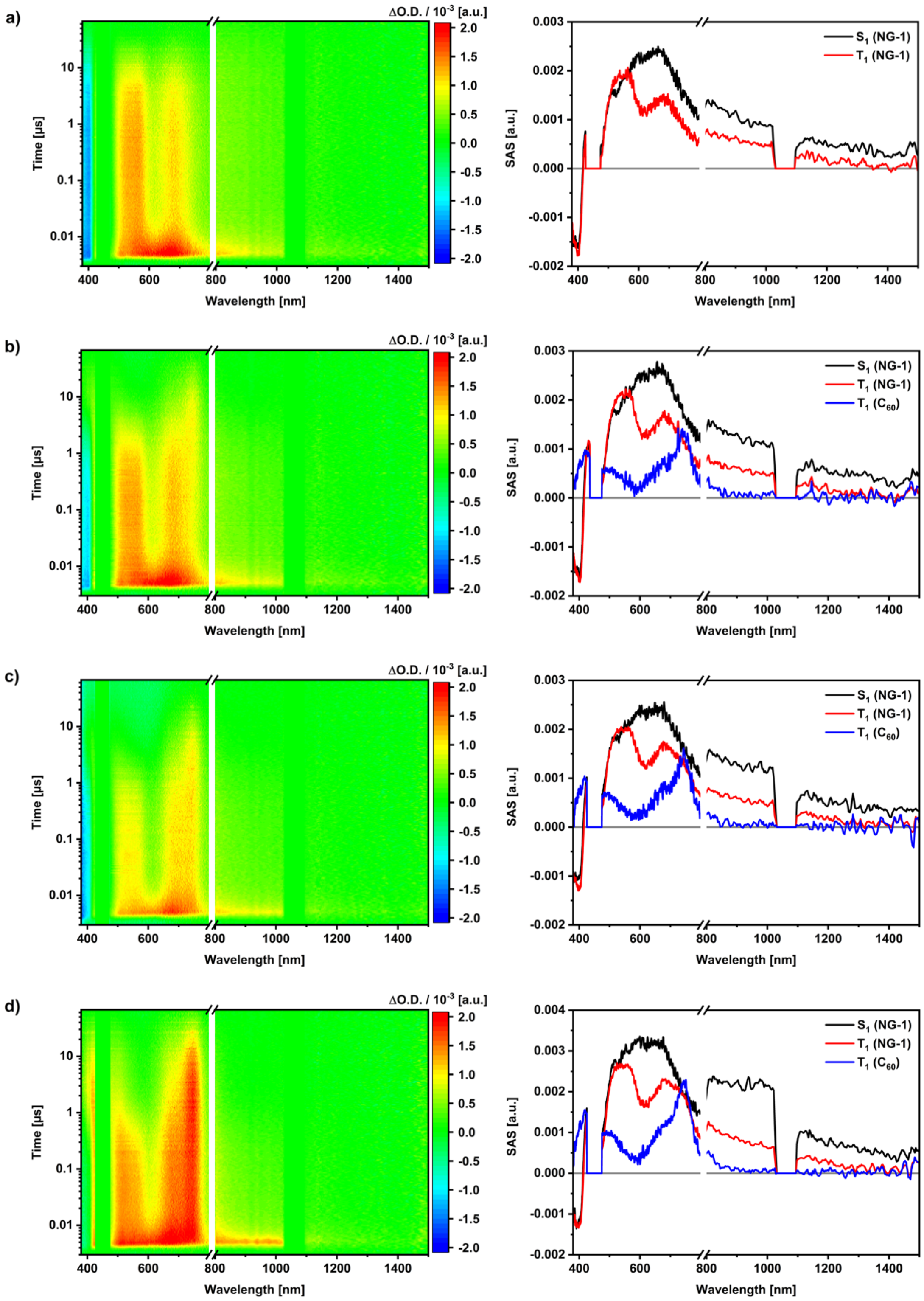

Figure S13. Differential transient absorption spectra of **NG-1** (1.0×10^{-5} M) and C_{60} in a) 1:0 b) 1:5 c) 1:10 and d) 1:25 molar ratio at time delays between 0 and 70 µs after laser excitation at 460 nm in argon purged chlorobenzene. SAS obtained from global target analysis are shown on the right: S_1(NG-1) in black, T_1(NG-1) in red, and T_1(C_{60}) in blue.

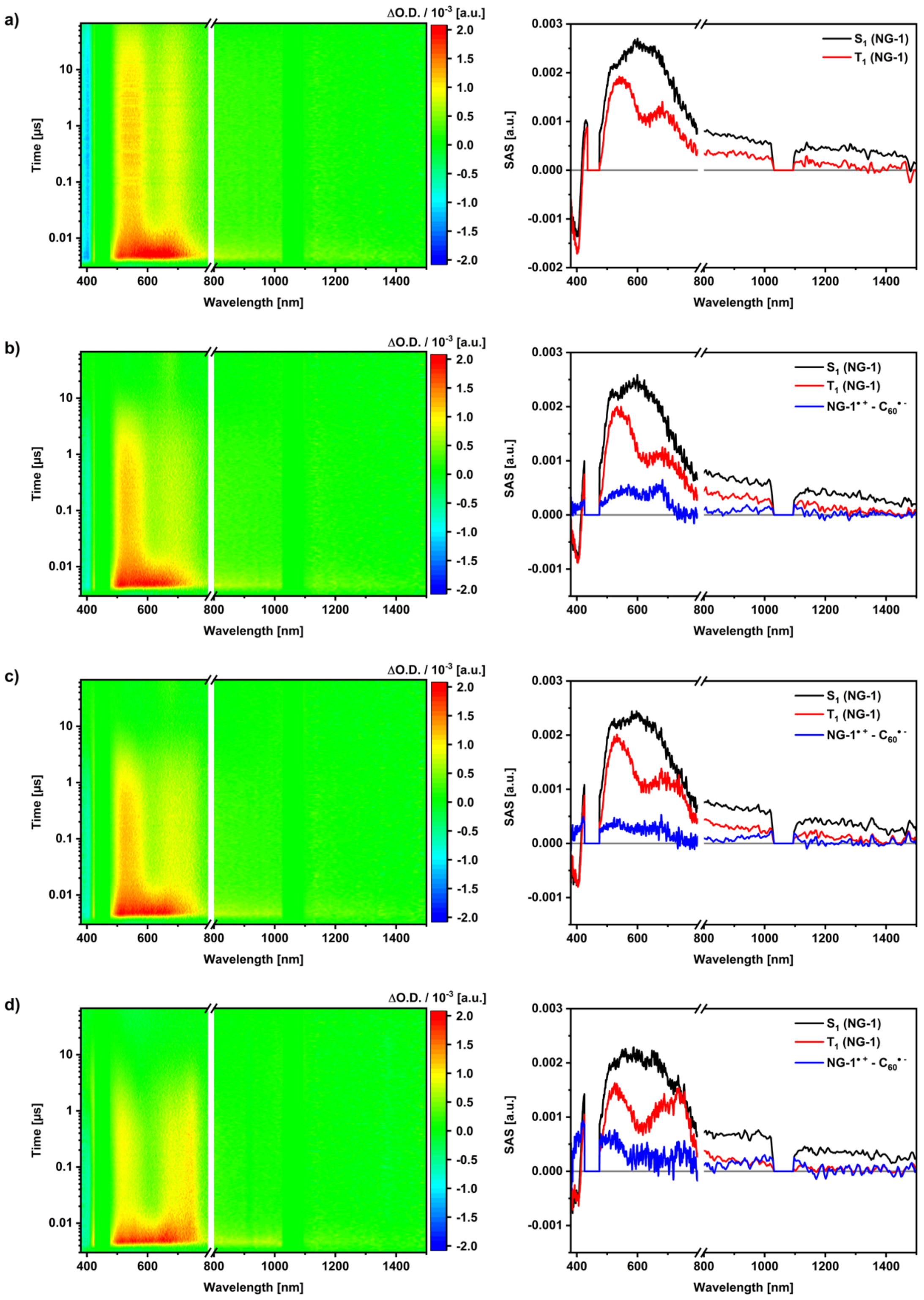

Figure S14. Differential transient absorption spectra of **NG-1** (1.0 x 10^{-5} M) and C_{60} in a) 1:0 b) 1:5 c) 1:10 and d) 1:25 molar ratio at time delays between 0 and 70 µs after laser excitation at 460 nm in argon purged benzonitrile. SAS obtained from global target analysis are shown on the right: S_1(NG-1) in black, T_1(NG-1) in red, and NG-$1^{•+}$ – $C_{60}{}^{•-}$ in blue.

5. References

1. J. M. Fernández-García, P. J. Evans, S. Medina Rivero, I. Fernández, D. García-Fresnadillo, J. Perles, J. Casado, N. Martín, *J. Am. Chem. Soc*. **2018**, *140*, 17188-17196.
2. P. Thordarson, *Chem. Soc. Rev.* **2011**, *40*, 1305-1323.
3. http://supramolecular.org last visited Jul 19, 2021
4. A. D. Becke, *Phys. Rev. A* **1988**, *38*, 3098-3100.
5. C. Lee, W. Yang and R. G. Parr, *Phys. Rev. B* **1988**, *37*, 785-789.
6. F. Weigend and R. Ahlrichs, *Phys. Chem. Chem. Phys.* **2005**, *7*, 3297-3305.
7. F. Weigend, *Phys. Chem. Chem. Phys.* **2006**, *8*, 1057-1065.
8. K. Eichkorn, O. Treutler, H. Öhm, M. Häser and R. Ahlrichs, *Chem. Phys. Lett.* **1995**, *240*, 283-290.
9. K. Eichkorn, F. Weigend, O. Treutler and R. Ahlrichs, *Theor. Chem. Acc.* **1997**, *97*, 119-124.
10. F. Neese, *WIREs Comput. Mol. Sci.* **2012**, *2*, 73-78.
11. ORCA—an ab initio density functional, and semiempirical program package, version 4.2.1
12. K. Eichkorn, F. Weigend, O. Treutler, R. Ahlrichs; *Theor. Chem. Acc.* **1997**, *97*, 119.
13. S. Hirata and M. Head-Gordon, *Chem. Phys. Lett.* **1999**, *314*, 291-299.
14. T. Yanai, D. P. Tew and N. C. Handy, *Chem. Phys. Lett.* **2004**, *393*, 51-57.
15. Gaussian 16, Revision A.03, M. J. Frisch, G. W. Trucks, H. B. Schlegel, G. E. Scuseria, M. A. Robb, J. R. Cheeseman, G. Scalmani, V. Barone, G. A. Petersson, H. Nakatsuji, X. Li, M. Caricato, A. V. Marenich, J. Bloino, B. G. Janesko, R. Gomperts, B. Mennucci, H. P. Hratchian, J. V. Ortiz, A. F. Izmaylov, J. L. Sonnenberg, D. Williams-Young, F. Ding, F. Lipparini, F. Egidi, J. Goings, B. Peng, A. Petrone, T. Henderson, D. Ranasinghe, V. G. Zakrzewski, J. Gao, N. Rega, G. Zheng, W. Liang, M. Hada, M. Ehara, K. Toyota, R. Fukuda, J. Hasegawa, M. Ishida, T. Nakajima, Y. Honda, O. Kitao, H. Nakai, T. Vreven, K. Throssell, J. A. Montgomery, Jr., J. E. Peralta, F. Ogliaro, M. J. Bearpark, J. J. Heyd, E. N. Brothers, K. N. Kudin, V. N. Staroverov, T. A. Keith, R. Kobayashi, J. Normand, K. Raghavachari, A. P. Rendell, J. C. Burant, S. S. Iyengar, J. Tomasi, M. Cossi, J. M. Millam, M. Klene, C. Adamo, R. Cammi, J. W. Ochterski, R. L. Martin, K. Morokuma, O. Farkas, J. B. Foresman, and D. J. Fox, Gaussian, Inc., Wallingford CT, **2016**.
16. S. Grimme, J. Antony, S. Ehrlich and H. Krieg, *J. Chem. Phys.* **2010**, *132*, 154104.
17. S. Grimme, S. Ehrlich and L. Goerigk, *J. Comput. Chem.* **2011**, *32*, 1456-1465.
18. R. S. Mulliken, *J. Chem. Phys.* **1955**, *23*, 1833-1840.
19. R. S. Mulliken, *J. Chem. Phys.* **1955**, *23*, 1841-1846.
20. P. O. Löwdin, *J. Chem. Phys.* **1950**, *18*, 365-375.
21. F. L. Hirshfeld, *Theor. Chim. Acta* **1977**, *44*, 129-138.
22. A. V. Marenich, S. V. Jerome, C. J. Cramer, D.G. Truhlar, *J. Chem. Theory Comput.* **2012**, *8*, 527-541.
23. E. R. Johnson, S. Keinan, P. Mori-Sánchez, J. Contreras-García, A. J. Cohen and W. Yang, *J. Am. Chem. Soc.*, **2010**, *132*, 6498-6506.
24. J. Contreras-García, E. R. Johnson, S. Keinan, R. Chaudret, J. Piquemal, D. Beratan and W. Yang, *J. Chem. Theory Comput.* **2011**, *7*, 625–632.
25. T. Lu, F. Chen, *J. Comput. Chem.* **2012**, *33*, 580-592.
26. G. A. Zhurko, Chemcraft 1.80 (build 523b) - graphical program for visualization of quantum chemistry computations. (https://chemcraftprog.com).
27. F. Plasser and H. Lischka, *J. Chem. Theory Comput.* **2012**, *8*, 2777-2789.
28. F. Plasser, S. A. Bäppler, M. Wormit and A. Dreuw, *J. Chem. Phys.* **2014**, *141*, 024107.
29. A. V.Luzanov and O.A. Zhikol, *Int. J. Quantum Chem.* **2010**, *110*, 902-924.
30. A.A. Voityuk and S.F. Vyboishchikov, *Phys. Chem. Chem. Phys.* **2019**, *21,* 18706-18713.

31. S.F. Vyboishchikov and A.A. Voityuk, *Phys. Chem. Chem. Phys.* **2020**, *22,* 14591-14598.
32. B. Mennucci, *WIREs Comput. Mol. Sci.*, **2012**, *2*, 386-404.
33. A. Klamt, G. Schüürmann, *J. Chem. Soc. Perkin Trans.* **1993**, *2*, 799–805.
34. J. Tomasi, B. Mennucci and R. Cammi, *Chem. Rev.* **2005**, *105*, 2999-3093.
35. J. L. Pascual-Ahuir, E. Silla, and I. Tuñón, *J. Comp. Chem.* **1994**, *15*, 1127–1138.
36. A. Klamt, *J. Phys. Chem.* **1996**, *100*, 3349-3353.
37. R. A. Marcus and N. Sutin, *Biochim. Biophys. Acta, Rev. Bioenerg.* **1985**, *811*, 265-322.
38. A. A. Voityuk and N. Rösch, *J. Chem. Phys.* **2002**, *117*, 5607-5616.
39. A. A. Voityuk, *Phys. Chem. Chem. Phys.* **2012**, *14*, 13789-13793.
40. T. Ziegler, A. Rauk, *Theor. Chim. Acta* **1977**, *46*, 1-10.
41. T. Ziegler, A. Rauk, *Inorg. Chem.* **1979**, *18*, 1558-1565.
42. T. Ziegler, A. Rauk*, Inorg. Chem.* **1979**, *18*, 1755-1759.
43. S. Grimme*, J. Comput. Chem.* **2004**, *25*, 1463-1473.
44. S. Grimme*, J. Comput. Chem.* **2006**, *27*, 1787-1799.
45. J. J. Snellenburg, S. Laptenok, R. Seger, K. M. Mullen, I. H. M. van Stokkum*, J. Stat. Softw.* **2012**, *43*, 1-22.
